# A Stable and Reproducible Human Blood-Brain Barrier Model Derived from Hematopoietic Stem Cells

**DOI:** 10.1371/journal.pone.0099733

**Published:** 2014-06-17

**Authors:** Romeo Cecchelli, Sezin Aday, Emmanuel Sevin, Catarina Almeida, Maxime Culot, Lucie Dehouck, Caroline Coisne, Britta Engelhardt, Marie-Pierre Dehouck, Lino Ferreira

**Affiliations:** 1 Blood Brain Barrier Laboratory, University of Artois, Lens, France; 2 CNC - Center of Neurosciences and Cell Biology, University of Coimbra, Coimbra, Portugal; 3 Biomaterials and Stem Cell-based Therapeutics Laboratory, Biocant - Center of Innovation in Biotechnology, Cantanhede, Portugal; 4 Institute for Interdisciplinary Research, University of Coimbra (IIIUC), Coimbra, Portugal; 5 Theodor Kocher Institute, University of Bern, Bern, Switzerland; Washington University, United States of America

## Abstract

The human blood brain barrier (BBB) is a selective barrier formed by human brain endothelial cells (hBECs), which is important to ensure adequate neuronal function and protect the central nervous system (CNS) from disease. The development of human *in vitro* BBB models is thus of utmost importance for drug discovery programs related to CNS diseases. Here, we describe a method to generate a human BBB model using cord blood-derived hematopoietic stem cells. The cells were initially differentiated into ECs followed by the induction of BBB properties by co-culture with pericytes. The brain-like endothelial cells (BLECs) express tight junctions and transporters typically observed in brain endothelium and maintain expression of most *in vivo* BBB properties for at least 20 days. The model is very reproducible since it can be generated from stem cells isolated from different donors and in different laboratories, and could be used to predict CNS distribution of compounds in human. Finally, we provide evidence that Wnt/β-catenin signaling pathway mediates in part the BBB inductive properties of pericytes.

## Introduction

BBB models can provide a valuable tool for studying mechanistic aspects related to the transport of drugs at the brain, as well as biological and pathological processes related to the BBB [1]. Although *in vitro* models were established from various species, the most widely used being rat, mouse, pig and bovine, the establishment of a stable human BBB model is very important to account for differences between species [1]. Primary human brain endothelial cells (hBECs) and immortalized human cells have been used as *in vitro* models [2], [3]; however, several issues prevent their general use including constraints in obtaining human tissue, loss of hBEC phenotype during immortalized cell culture, or lack of important tight junctions and low transendothelial electrical resistance (TEER) values as shown in human cell lines. Recently, hBECs have been differentiated from induced pluripotent stem cells (iPSCs) [4]. However, the reproducibility of paracellular permeability and TEER across replicates was relatively low. In addition, it is unclear whether the reproducibility of the model is affected by the type and history of iPSC line used to derive the hBECs and the stability of the *in vitro* BBB model for periods of time above 7 days, which might preclude its general use for drug screening and toxicology studies [4]. Also recently, a human *in vitro* BBB model based on the co-culture of cord blood-derived ECs with astrocytes has been reported [5]. However, the BBB model presents low TEER values and relatively high permeability (e.g. Pe to Lucifer yellow = 1.23×10^−3 ^cm/min).

Here, we report a general and relatively easy method to generate a human BBB model using cord blood-derived hematopoietic stem cells, which can be obtained non-invasively. The cells were initially differentiated into endothelial cells (ECs) followed by the induction of BBB properties by co-culture with pericytes. The model is very reproducible (similar paracellular permeability for cells derived from 3 different donors and in 3 different laboratories) and stable (for at least 20 days). Our results show for the first time a good correlation between the *in vitro* predicted ratio of concentrations of unbound drug in brain and plasma obtained with our model and the *in vivo* ratio of concentrations of unbound drugs in cerebrospinal fluid (CSF) and plasma reported in humans. Finally, we show that Wnt signalling pathway mediates in part the BBB inductive properties of pericytes.

## Materials and Methods

An expanded version of the Methods section is provided in Text S1. Materials and Methods.

### Isolation and Differentiation of CD34^+^ Cells from Human Umbilical Cord Blood (UCB)

All human UCB samples were collected from donors, who signed an informed consent form, in compliance with Portuguese legislation. The collection was approved by the ethical committees of Dr. Daniel de Matos Maternity Hospital in Coimbra and Hospital Infante D. Pedro in Aveiro. CD34^+^ cells were isolated from human UCB and differentiated into ECs according to a protocol previously reported by us [6]. Briefly, isolated CD34^+^ cells were cultured in EGM-2 medium (Lonza) supplemented with 20% (v/v) fetal bovine serum (FBS; Life Technologies) and 50 ng/mL of VEGF_165_ (PeproTech Inc.), on 1% (w/v) gelatin-coated 24-well plates (2×10^5^ cells/well). After 15–20 days ECs are seen in the culture dish. For each experiment, the cells were expanded in 1% (w/v) gelatin-coated 100 mm Petri dishes (BD Falcon) in EGM-2 medium (with all the supplements except FBS and gentamycin/amphotericin) supplemented with 2% (v/v) FBS, 50 µg/mL gentamycin (Biochrom AG) and 1 ng/mL basic fibroblast growth factor (bFGF).

### Co-culture Experiments

For co-culture experiments, pericytes were initially seeded on 60-mm gelatin-coated petri dishes and cultured in Dulbecco’s Modified Eagle’s Medium (DMEM) (Life Technologies) supplemented with 20% (v/v) FBS (Life Technologies), 2 mM L-glutamine, 50 µg/mL gentamycin and 1 ng/mL bFGF. The cells reached confluency after 2 days. 45×10^3^ cells were seeded into each well of 12-well plates (Costar). CD34^+^-ECs growing on gelatin-coated 100 mm petri dishes in EGM-2 (with all the supplements except FBS and gentamycin/amphotericin) supplemented with 2% (v/v) FBS, 50 µg/mL gentamycin (Biochrom AG) and 1 ng/mL bFGF were trypsinized and cells were seeded at a density of 8×10^4^/insert onto the Matrigel-coated (BD Biosciences) Transwell inserts (Costar).

### Endothelial Permeability (Pe) Measurements

Prior to the experiments, HEPES-buffered Ringer’s solution (in some cases EBM-2 medium) was added to empty wells of a 12-well plate (Costar). Filter inserts, containing confluent monolayers of CD34^+^-ECs, were subsequently placed in the 12-well plate, filled with compound solution containing the fluorescent integrity marker Lucifer Yellow (20 µM; Life Technologies), and then placed on an orbital shaker. After 1 h, filter inserts were withdrawn from the receiver compartment. Aliquots from the donor solution were taken at the beginning and at the end of the experiments and the fluorescence was quantified. At least three inserts with cells and three without cells were tested in each permeability measurement. Fluorescence detection was carried out on a Synergy H1 multiplates reader (Biotek) using the following excitation/emission wavelength (nm) settings: 432/538; 490/516; 542/570 for Lucifer yellow, Fluorescein Na and Cy3-Human Serum Albumin and Human IgG respectively.

To obtain a concentration-independent transport parameter, the clearance principle was used. The increment in cleared volume was calculated by dividing the amount of compound in the receiver compartment by the drug concentration in the donor compartment [7]. The volume cleared was plotted versus time and the slope estimated by linear regression analysis. The slope of the clearance curve with inserts alone and inserts with cells is equal to PSf and PSt, respectively, where PS (microliters/minute) is the permeability surface area (square centimeter) product. The PS-value for endothelial monolayer (PSe) was calculated. To generate the endothelial permeability coefficient, Pe (cm/min), the PSe value was divided by the surface area of the filter (A in cm^2^) insert using the following equation: Pe = [1/PSt−1/PSf]^−1^/A. To assess possible adsorption to plastics and non-specific binding to cells, the mass balance (%) was calculated from the amount of compound recovered in both compartments at the end of the experiment divided by the total amount added in the donor compartment at time zero. For Pe determination, mass balance value should be between 80% and 120%.

### Wnt Signaling Experiments

For Wnt signaling experiments, mono- and co-culture systems were used. In monoculture, 8×10^4^ CD34^+^-ECs were seeded on the Matrigel-coated transwell insert. The cells were then incubated with agonists/ligands (6.25 ng/mL–100 ng/mL Wnt3A (R&D Systems), 6.25 ng/mL–250 ng/mL Wnt7A (Peprotech) or 0.5–5 µM BIO (Sigma)) for 1 or 5 days. Co-cultures were prepared as described before. The CD34^+^-derived ECs co-cultured with pericytes for 1 or 6 days were used in the signaling experiments. Agonist (0.5–5 µM BIO) was added into the basolateral compartment while antagonist (0.1–3 µM XAV939 (Selleckbio)) was added in the apical part of the transwell system.

### Expression of Adhesion Molecules by BLECs

Adhesion molecule expression by BLECs was determined by FACS. For these experiments, CD34^+^-ECs were cultured with pericytes for 6 days. After co-culture, transwells with BLEC monolayers were transferred to a new 12-well plate. BLECs were treated with 10 ng/mL TNF-α (Peprotech) for 24 h. Untreated BLECs were used as control. Cells were dissociated from the culture plate by exposure to Cell Dissociation Buffer (Life Technologies) for 3–5 min and gentle pipetting, centrifuged and finally resuspended in PBS supplemented with 5% (v/v) FBS. The single cell suspensions were aliquoted (2.0×10^5^ cells per condition) and incubated with primary antibodies against human CD40, ICAM1, ICAM2, VCAM1, PECAM1 (**Table S1**). After the incubation with primary antibodies, cells were incubated with phycoerytrin (PE)-conjugated anti-rabbit (R&D Systems), and PE-conjugated anti-mouse (Santa Cruz) secondary antibodies. FACS Calibur (BD Biosciences) and BD Cell Quest Software (BD Biosciences) were used for the acquisition and analysis of the data.

### Ultrastructural Analysis of Cell Monolayers by Transmission Electron Microscopy (TEM)

Wheat germ agglutinin conjugated horseradish peroxidase (WGAHRP) (Sigma-Aldrich) was used for ultrastructural analysis of EC monolayers. Filter inserts with ECs were transferred into plates containing 1.5 mL of HEPES-buffered Ringer’s solution (150 mM NaCl, 5.2 mM KCl, 2.2 mM CaCl_2_, 0.2 mM MgCl_2_-6H_2_O, 6 mM NaHCO_3_, 5 mM HEPES, 2.8 mM glucose, pH 7.4) (lower compartment), and 0.5 mL of HEPES-buffered Ringer’s solution supplemented with 0.1 mg/mL WGA-HRP was applied to the upper compartment. After 10 min incubation at 37°C in a 5% CO_2_/95% air atmosphere, the WGA-HRP solution was removed and the specimens were washed twice with HEPES-buffered Ringer’s solution and fixed for 1 h at room temperature with 2.5% glutaraldehyde and 1% paraformaldehyde in 0.1 M sodium cacodylate (pH 7.4). After washing with 0.1 M sodium cacodylate, the fixed EC monolayers were incubated for 30 min at room temperature with the HRP substrate 3, 3′-diaminobenzidine tetrahydrochloride (1.5 mg/mL; Sigma-Aldrich) and 0.02% H_2_O_2_ (v/v) in a TRIS-imidazol buffer (0.1 M imidazol, 0.05 M TRIS/HCl, pH 7.0). After washing with 0.1 M sodium cacodylate, cells were fixed again for 1 h at room temperature with 2.5% glutaraldehyde and 1% paraformaldehyde in cacodylate buffer. Specimens were washed twice with 0.1 M sodium cacodylate buffer, postfixed with 1% OsO4 in 0.1 M cacodylate buffer. After dehydration in graded ethanol, samples were embedded in Epon 812. Ultrathin sections were cut on Ultracut UCT (Leica), contrasted with uranyl acetate and lead citrate, and examined with a Jeol 1011 TEM at an accelerating voltage of 100 Kv.

### Microarray Studies

CD34^+^-ECs were cultured in monoculture or in co-culture with pericytes for 3 days and 6 days in the same culture conditions described in the CD34^+^-ECs co-culture experiments section. At days 3 and 6, the CD34^+^-ECs were homogenized in Trizol reagent (Life Technologies) and the total amount of RNA was extracted with RNeasy Mini Kit (Qiagen), according to manufacturer’s instructions. RNA quality was assessed by an Agilent 2100 Bioanalyser (G2943CA), using an Agilent RNA 6000 Nano Kit (5067–1511). Gene expression was evaluated by a whole human genome (4×44 K) microarray (G4112F from Agilent Technologies). The microarrays were scanned by an Agilent B Scanner (G2565BA). The raw data were analyzed using BRB-ArrayTools v3.4.0 developed by Dr. Richard Simon and BRB-ArrayTools Development Team [8]. This analysis generated a median normalized dataset that was subjected to a statistical study and clustering using MeV software [9]. The differential expressed genes obtained from MeV were used to calculate the M-value and Fold-change variation. It was considered as differentially expressed gene a variation equal or higher than 2 times between the different conditions. The microrray data in this paper have been deposited in GEO (Gene Expression Omnibus) (accession no. GSE45171). The results of microarray were confirmed by qRT-PCR (**Text S1** and **Table S2)**.

### Statistical Analysis

For analysis involving three or more groups, ANOVA was used, followed by a Bonferroni post-test. For analysis of two groups, a paired *t*-test was used. Statistical analysis was performed using GraphPad Prism software (San Diego, CA, USA). Results were considered significant when *P*≤0.05.

## Results

### Cord Blood Stem Cells can Differentiate into Brain-like ECs

To differentiate stem cells into ECs, CD34^+^CD45^+^CD31^+^KDR^−^vWF^−^CD14^−^ cells isolated from cord blood were initially cultured for 15–20 days in EGM-2 medium with 20% (v/v) FBS and 50 ng/mL of VEGF_165_ [6] (**Fig. S1A**). At this stage, cells have a cobblestone-like morphology, express high levels of EC markers, including CD31, VE-cadherin and vWF and are able to incorporate Ac-LDL (**Fig. S1B**). When these cells were grown to confluence on filters for 6 days they show discontinuous expression of ZO-1, occludin and claudin-5, do not express claudin-1 at cell-cell contacts and have high permeability to Lucifer yellow (2.0×10^−3 ^cm/min) as compared to bovine BECs (**Figs. S1C and S1D**).

**Figure 1:**
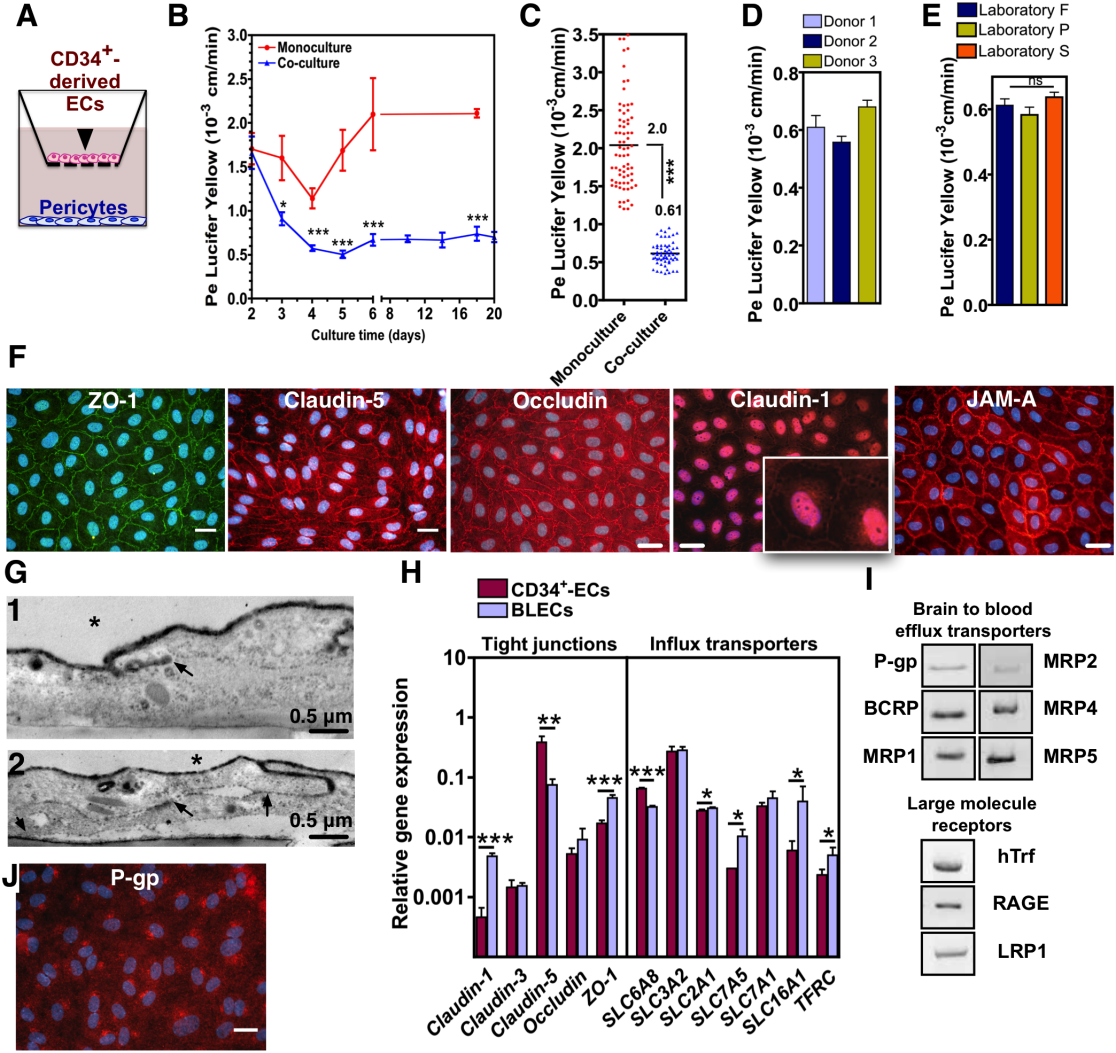
Expression of BBB markers, stability, reproducibility and functional properties of a monolayer of human BLECs. (A) BLECs were obtained by the co-culture of CD34^+^-derived ECs with pericytes for 6 days in a transwell system. (B–C) Paracellular permeability to lucifer yellow of EC monolayers either cultured alone or with pericytes. (D–E) Paracellular permeability in a co-culture of CD34^+^-derived ECs with pericytes at day 6 obtained from (D) different donors and at (E) different laboratories (P = Portugal (LF); F = France (RC); S = Switzerland (BE)). From B to E, results are Mean ± SEM (n≥4). (F) Expression of BBB markers in BLECs as obtained by immunofluorescence. (G) Electron micrographs of ECs co-cultured with pericytes for 6 days (1) or alone (2). (1) Occlusion of the intercellular space between the ECs. WGA-HRP penetrates partially the intracellular cleft (arrow). (2) No occlusion of the intercellular space between the ECs in 84% of the cases. WGA-HRP penetrates from the luminal compartment (asterisk) through the entire intercellular cleft and is deposited in the underlying matrix (arrows). (H) Gene expression of tight junctions and influx transporters in BLECs and CD34^+^-derived ECs. Results are Mean ± SEM (n = 3). (I) Gene expression of efflux transporters and large molecule receptors in BLECS, i.e., CD34^+^-derived ECs co-cultured with pericytes for 6 days. β actin was used as housekeeping gene (J) Expression of P-gp as evaluated by immunofluorescence. In G and K, bar corresponds to 50 µm. *P<0.05, **P<0.01, ***P<0.001.

To induce BBB properties in CD34^+^-derived ECs, cells were seeded in a transwell system and co-cultured with pericytes (**Fig. 1A**). Pericytes were selected after a screening of different cell types from the neurovascular unit (**Figs. S2A and S2B**) and because of their role in the stabilization/maturation of BBB [10], [11]. Under these conditions, the permeability of ECs decreases during the first 3 days until it reaches a stationary phase at day 4, maintaining its stability up to 20 days (**Fig. 1B**). At day 6, the cells had low permeability to Lucifer yellow values (0.61±0.15×10^−3 ^cm/min, *n* = 60) similarly to the values found in other BBB models [12] (**Fig. 1C**), they showed a continuous expression of ZO-1, occludin, JAM-A, claudin-1 and claudin-5 at cell-cell contacts (**Fig. 1F**) and they were able to block the passage of wheat germ agglutinin (WGA)- horseradish peroxidase (HRP) in contrast with monolayers of CD34^+^-derived ECs where WGA-HRP reached the underlying matrix (**Fig. 1G**). Importantly, the induction of BBB properties in CD34^+^-derived ECs is highly reproducible since similar permeability results were obtained for cells derived from multiple human donors (**Fig. 1D**) and in 3 different laboratories (**Fig. 1E**). Furthermore, the BBB properties of CD34^+^-derived ECs require the presence of pericytes, since pericyte-conditioned medium does not have the same BBB-inductive properties, and are lost if the pericytes are removed from the co-culture system (**Figs. S2C and S3B**). These results show that the crosstalk between the two cells is important to maintain the BBB properties. Cells co-cultured with pericytes for 6 days express transcripts encoding tight junctions such as ZO-1 and claudin-1 higher than in ECs in monoculture, express claudin-3 and occludin at similar levels as ECs in monoculture, and express claudin-5 at lower levels as EC in monoculture (**Fig. 1H**). Importantly, the expression of influx transporters, specifically the expression of aminoacid (SLC7A5, SLC16A1) and glucose (SLC2A1) transporters and receptors (e.g. transferrin receptor; TFRC) was increased when the cells were co-cultured with pericytes relatively to cells cultured alone. The results are consistent with previous results showing that the induction of BBB properties in ECs correlate with an up-regulation of specific transporter systems, most prominently SLC2A1 (Glut-1) [4], [13]. In addition, ECs co-cultured with pericytes for 6 days express transcripts of key efflux transporters such as P-glycoprotein (P-gp), breast cancer resistance protein (BCRP) and multidrug resistance protein (MRP; subfamily of the ATP-binding cassette (ABC) transporters) family, and they express large molecule receptors such as low-density lipoprotein receptor-related protein 1 (LRP1), the receptor for advanced glycation end products (RAGE) and transferrin receptor (hTrf) (**Fig. 1I**). The expression of RAGE, organic cation/carnitine transporter (OCTN2; also known as SLC22A5) (**Fig. S3A)** and P-gp protein (**Fig. 1J**) was further confirmed by immunofluorescence. As in hBECs, RAGE is mainly located at the luminal side of cells while OCTN2 is located at the abluminal side. Overall, ECs co-cultured with pericytes for 6 days have BBB properties at gene, protein and permeability levels, and from now on are named as brain-like endothelial cells (BLECs).

### BLECs have the Ability to Act as an Active Barrier

**Figure 2:**
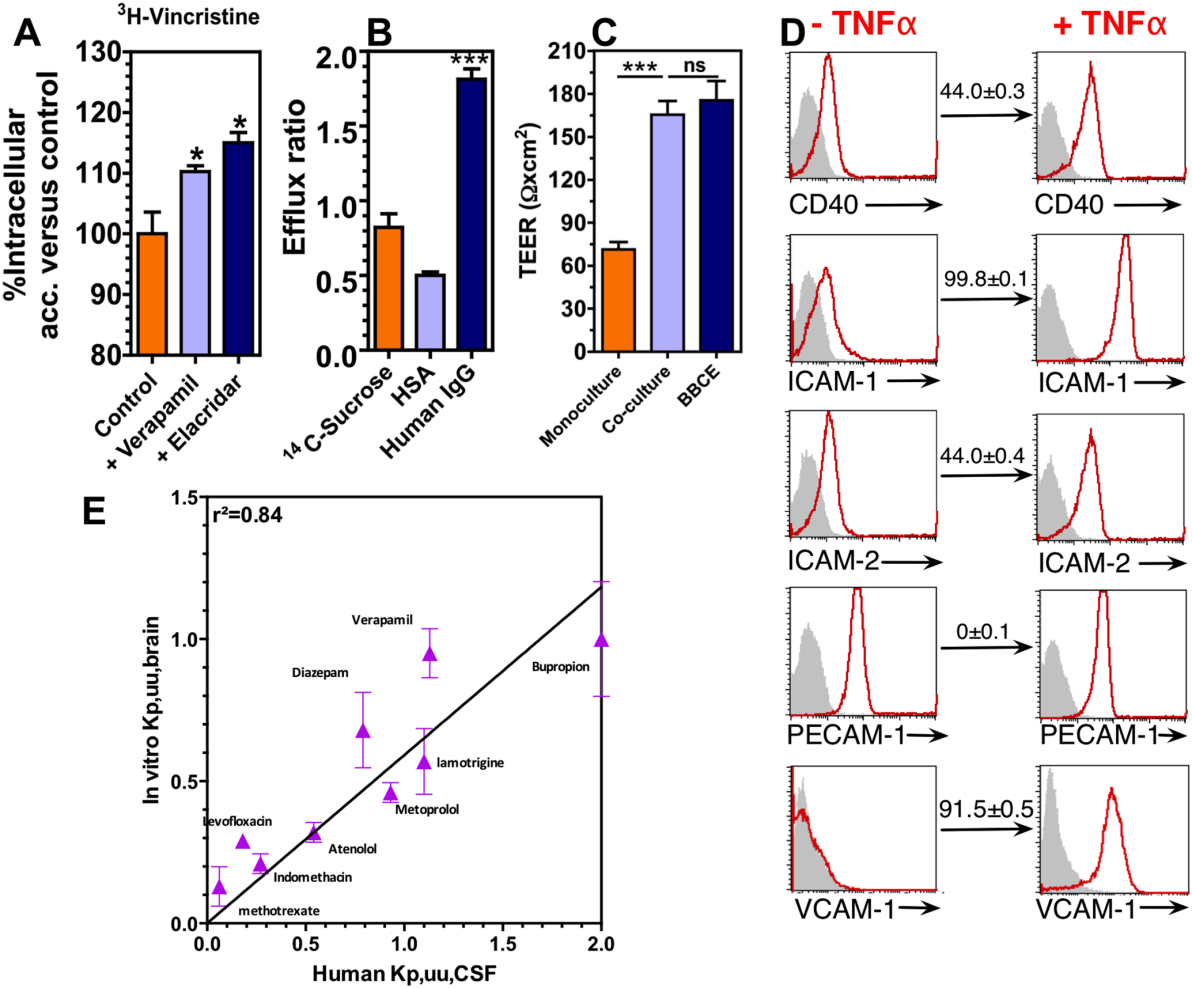
Functional properties of BLECs. (A) Effect of P-gp protein inhibition on active transport of drugs. (B) Efflux ratio of small (sucrose) and large (HSA and IgG) molecules. In A and B: Mean ± SEM (n = 3–7). (C) Transendothelial electrical resistance (TEER) of monocultures of CD34^+^-derived ECs or co-cultures of ECs with pericytes for 6 days. The TEER of the co-culture of ECs was compared with the gold standard of bovine brain microvascular endothelial cells co-cultured with bovine astrocytes for 12 days on insert filters 30 mm diameter. Values are Mean ± SEM, n = 4. ***P<0.001; ns means *P*>0.05. (D) Expression of adhesion molecules by ECs in co-culture with pericytes. The expression of the adhesion molecules was assessed by flow cytometry analysis on untreated and treated ECs by TNFα (10 ng/mL) for 24 h. (E) Correlation (r^2^ = 0.84; Pearson r = 0.9160) between our human *in vitro* data (K_p,uu,brain_) and human K_p,uu,CSF_ data (obtained from ref. [18]). K_p,uu,CSF_ = (*In vivo* concentration of unbound drug in cerebrospinal fluid (CSF))/(*In vivo* concentration of unbound drug in plasma). K_p,uu,brain_ = (*In vitro* concentration of unbound drug in brain)/(*In vitro* concentration of unbound drug in plasma). K_p,uu,brain_ were calculated from flux experiments using triplicate filters as described in Text S1. Materials and Methods. Values are Mean ± SEM (n = 3).

The inhibition of P-gp protein by verapamil or elacridar, and the concomitant blocking of the active transport of drugs to outside the cell [14], leads to a significant increase in the accumulation of the antitumor drug vincristine (**Fig. 2A**). This result demonstrates that P-gp is functionally active in BLECs. The higher efflux ratio of IgG as compared to human serum albumin shows receptor-mediated transport of this macromolecule across the polarized monolayer from the abluminal to the luminal side (**Fig. 2B**). In addition, BLECs have the ability to form a monolayer that has a TEER similar to monolayers of bovine BECs (**Fig. 2C**) and higher than monolayers of human hCMEC/D3 cell line (<40 Ω×cm^2^) [2] or monolayers of cord blood-derived ECs co-cultured with astrocytes [5]. Moreover, BLECs express constitutively the adhesion molecule PECAM-1 and ICAM-2, typically found in hBECs [15]. These molecules are up-regulated in ECs exposed to tumor necrosis factor alpha (TNF-α) mediated by the activation of the pleiotropic nuclear factor–kB (NF-kB) [16]. Accordingly, BLECs show an up-regulation in the expression of ICAM-1, ICAM-2, CD40 and VCAM-1 after stimulation with 10 ng/mL TNF-α for 24 h, as hBECs [2] (**Fig. 2D**). Taken together, these results show that our *in vitro* model is functional and can reproduce key aspects of the human BBB activity.

Importantly, BLECs can be used to predict CNS distribution in humans of drugs with different properties. As the unbound brain-to-plasma concentration ratio (K_p,uu,brain_) is considered as a major pharmacokinetic parameter in drug discovery [17], [18] we recently developed a methodology for assessing this parameter using an *in vitro* model of the BBB [19]. Thus, the ability of BLECs to assess unbound brain/unbound plasma concentration ratio in human has been evaluated and the data obtained were compared to the ratio of unbound CSF-to-plasma concentration (K_p,uu,CSF_) in human, which is frequently used as a surrogate measure of K_p,uu,brain_ [18]. For the 9 compounds tested, the estimation given by our *in vitro* model for K_p,uu,brain_ correlates well (r^2^ = 0.89) with K_p,uu,CSF_ in humans taken as a surrogate measurement the unbound brain-to-plasma concentration ratio (K_p,uu,brain_) (**Fig. 2E** **; Text S1.** **Materials and Methods**).

### The Induction of BBB Properties in ECs by Pericytes Involves Changes in Gene Expression of Wnt Signaling, Tight Junctions and Transporters

**Figure 3:**
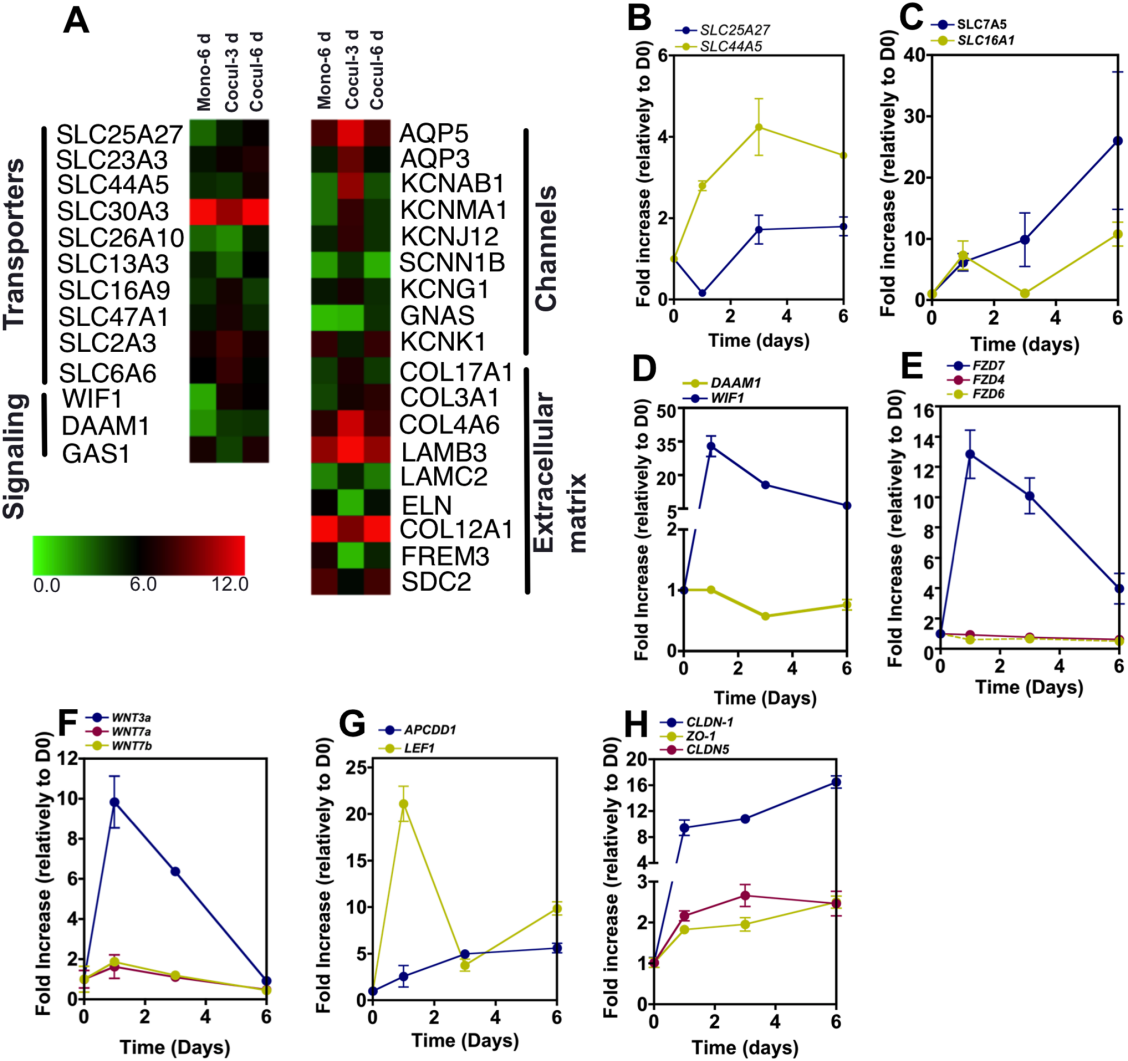
Gene expression during the induction of BBB properties in CD34^+^-derived ECs. (A) Expression of BBB markers as evaluated by whole genome microarrays of monocultures or co-cultures of CD34^+^-derived ECs with pericytes at day 3 and 6. (B–H) qRT-PCR results showing changes on BBB transporters (B, C), Wnt signaling (D–G) and tight junctions (H) genes on CD34^+^-derived ECs co-cultured with pericytes for 1, 3 and 6 days. Values are Mean ± SEM, n = 4.

To study the induction of BBB properties in CD34^+^-derived ECs, ECs cultured alone or with pericytes for 3 or 6 days were characterized by whole genome microarrays. Gene expression analyses at 6 days show that 84 and 2 genes are up- and down-regulated in CD34^+^-derived ECs in co-culture, respectively, relatively to CD34^+^-derived ECs in monoculture (**Tables S3 and S4**). From the overall up-regulated genes, 3 genes were related with influx transporters including SLC44A5, SLC25A27 and SLC23A3 (**Fig. 3A**). SLC44A5, SLC25A27 genes were further confirmed by qRT-PCR (**Fig. 3B**). Both genes peaked at day 3. Although not shown in the microarray data, the expression of other transporters (SLC7A5 and SLC16A1) in ECs cultured with pericytes was statistically different to ECs cultured alone by qRT-PCR (**Fig. 1H**). In this case, the temporal expression of the transporters SLC7A5 and SLC16A1 genes peaked at day 6, as revealed by qRT-PCR (**Fig. 3C**). These results show that the induction of BBB properties in ECs involves the up-regulation of several transporters.

Two major pathways regulating the formation of BBB are the canonical Wnt/wingless pathway acting via β-catenin stabilization and Sonic hedgehog (Shh) pathway [13], [20], [21]. Two genes related with Wnt signaling (Wnt inhibitory factor 1 (Wif1) and disheveled associated activator of morphogenesis 1 (Daam 1)) were up-regulated as shown by the whole genome microarray, suggesting the involvement of Wnt signaling in the formation of the BBB (**Fig. 3A**). The expression of those genes was monitored overtime by qRT-PCR. Daam1 expression slightly decreased after day 3, while Wif1 expression peaked at day 1 and then decreased until day 6 (**Fig. 3D**).

**Figure 4:**
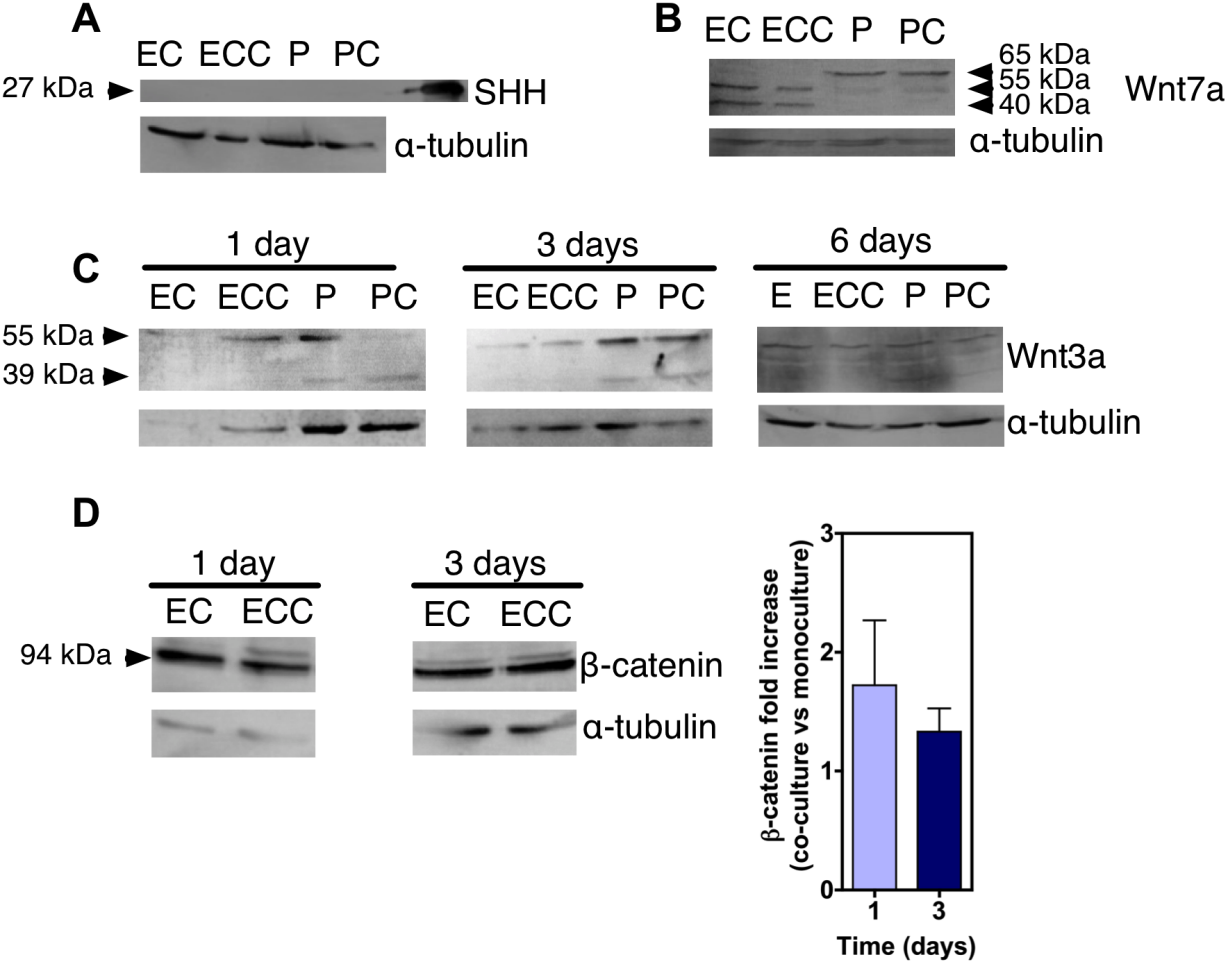
Expression of Shh, Wnt7a and Wnt3a in pericytes and CD34^+^-derived ECs in mono-culture or in co-culture. (A–D) Western blot for the expression of Shh (A), Wnt7a (B), Wnt3a (C) and total β-catenin (D) on cell lysates of CD34^+^-derived ECs in monoculture (E) or in co-culture with pericytes (ECC), or cell lysates of pericytes in monoculture (P) or pericytes in co-culture with ECs (PCC), for 6 days. Human recombinant Wnt3a, Wnt7a and Shh were used as a positive control. Data shown are representative of *n* = 2. In D: results ± SEM, *n* = 2.

We further investigated the involvement of Wnt signaling in the BBB specification by analyzing the expression of Wnt ligands and receptors in pericytes and ECs, respectively. Our protein analyses show that pericytes do not express Shh but do express Wnt ligands such as Wnt3a and Wnt7a (**Fig. 4**). On the other hand, ECs express at the mRNA level Wnt receptors such as frizzled receptor 4, 6 and 7 (FZD4, FZD6 and FZD7) (**Fig. 3E**). Once in co-culture with pericytes, ECs show a significant increase in Wnt3a transcript at day 1 followed by a decrease at day 6 to baseline levels (**Fig. 3F**); an increase of canonical Wnt ligands Wnt7a and Wnt7b transcripts, which have been reported to be involved in BBB development [21], [22], at day 1 followed by a decrease at day 3 to baseline levels (**Fig. 3F** **)**; and an increase in Wnt receptor frizzled 7 (FZD7) transcripts up to 6 days, but not in Wnt receptor frizzled 4 (FZD4) and frizzled 6 (FZD6) (**Fig. 3E**). The expression of LEF1, the β-catenin-associated transcription factor, peaked at day 1 matching the profile observed for Wnt3a and FZD7 (**Fig. 3G**). The expression of APCDD1, an antagonist of Wnt signaling and highly expressed in adult brain endothelial cells [23], peaked at day 3, at the time that Wnt3a drops significantly (**Fig. 3G**). We also investigated the temporal expression of tight junctions in ECs co-cultured with pericytes since our results (**Fig. 1H**) indicated a statistical difference in the expression of ZO-1, claudin-5 and claudin-1 in ECs cultured alone or co-cultured with pericytes. qRT-PCR results show an up-regulation of claudin-1 transcripts for 6 days in ECs cultured with pericytes while the expression of ZO-1 and claudin 5 transcripts peaked at day 6 and 3, respectively (**Fig. 3H**). Overall, our results indicate that the induction of BBB properties in ECs by pericytes involves, at least in part, Wnt signaling, and an increase in the expression of tight junctions ZO-1, claudin-5 and claudin-1, and several transporters.

### Wnt3a and in Minor Extent Wnt7a Mediate in Part the Induction of BBB Properties in ECs by Pericytes

**Figure 5:**
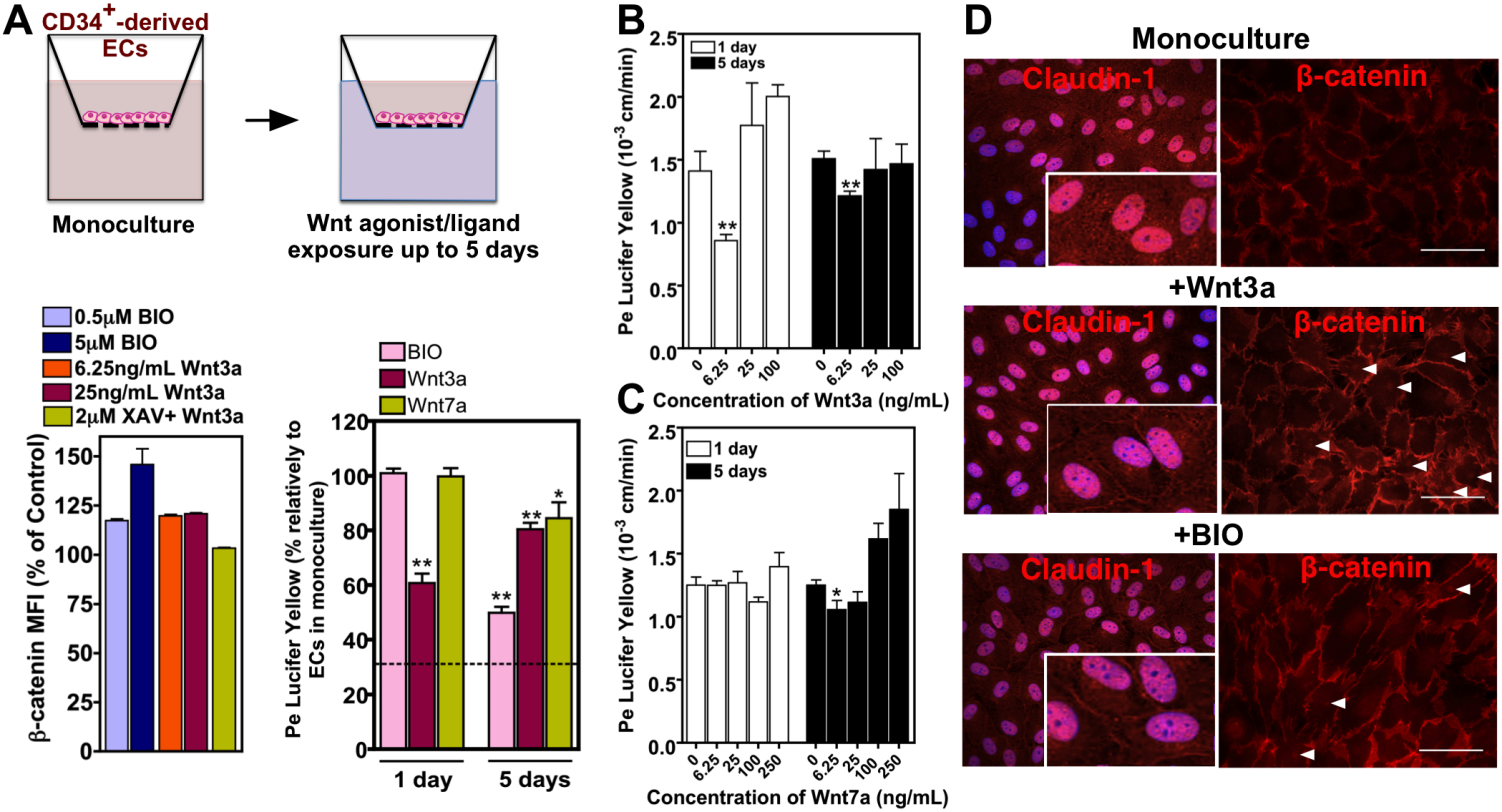
Activation of Wnt signaling in ECs by pericytes mediates BBB formation. (A) Effect of Wnt3a, Wnt7a and BIO in the expression of β-catenin (after 1 day) as well as in the paracellular permeability (at days 1 and 5) of monocultures of CD34^+^-derived ECs. Results are Mean ± SEM (n = 3–6). The dashed line represents the paracellular permeability of ECs in co-culture with pericytes for 6 days. For permeability results the concentrations of Wnt3a, Wnt7a and BIO were 6.25 ng/mL, 6.25 ng/mL, and 0.5 µM. (B–C) Paracellular permeability of untreated ECs or ECs treated with different concentrations of human recombinant protein Wnt3a (B) or Wnt7a (C) for 5 days. Results are Mean ± SEM (n = 4). (D) Expression and localization of claudin-1 (at day 6) and total β-catenin (day 3) in monoculture of CD34^+^-derived ECs cultured in medium supplemented with BIO (0.5 µM) or Wnt3a (6.25 ng/mL). Arrowheads indicate nuclear accumulation of β-catenin. Bar corresponds to 50 µm. *P<0.05, **P<0.01, ***P<0.001.

**Figure 6:**
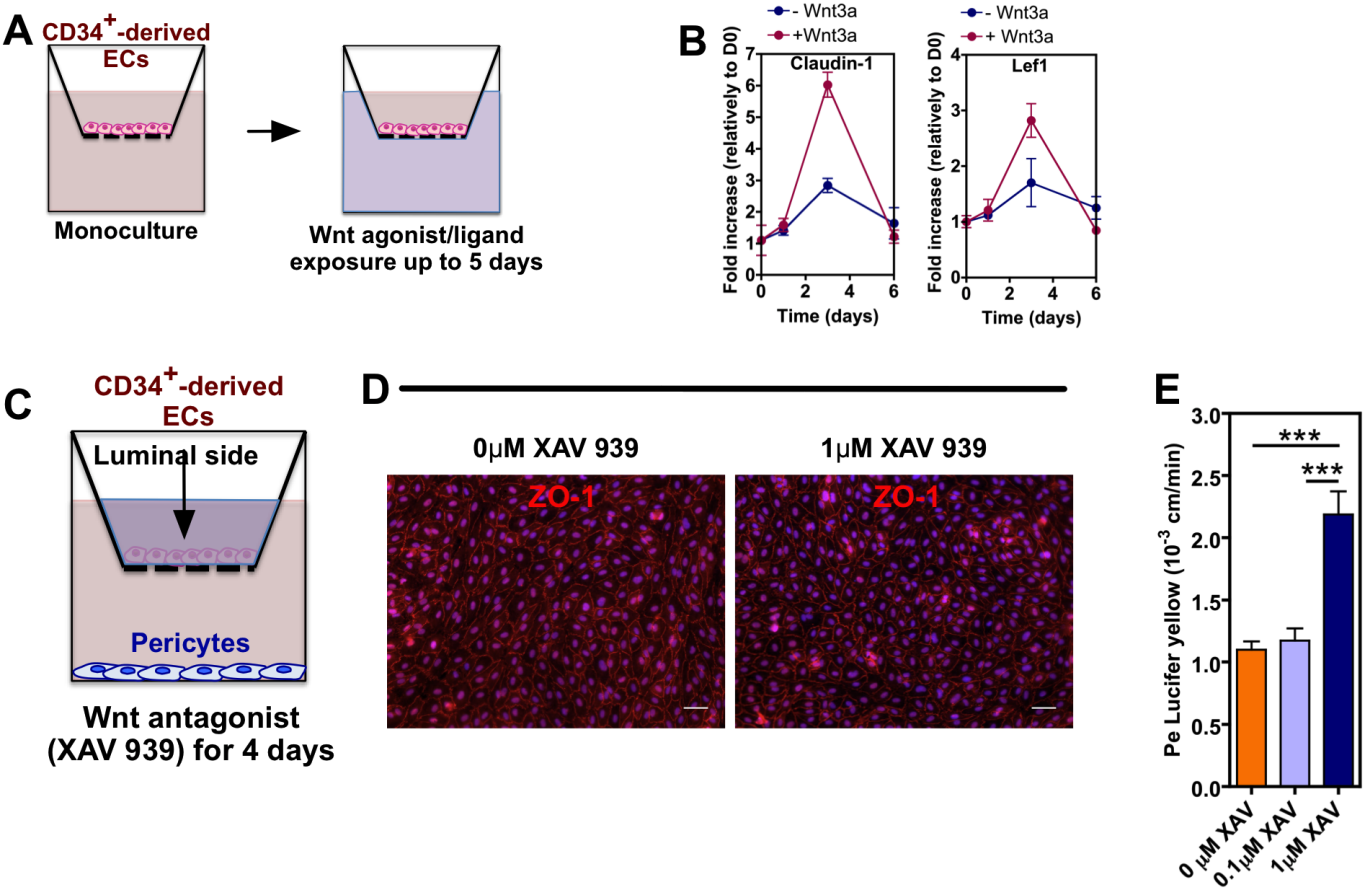
Evaluation of Wnt signaling in the induction of BBB properties in ECs. (A) Schematic representation of the methodology used to assess the modulation of Wnt signaling. CD34^+^-derived ECs were seeded in a transwell insert coated with Matrigel at a density of 80,000 cells. Wnt ligands were added in the culture medium at the basolateral side. (B) qRT-PCR results showing differences in expression of claudin-1 and Lef1 genes on CD34^+^-derived ECs cultured with or without Wnt3a. Values are Mean ± SEM, *n* = 4. (C) Schematic representation of the methodology used to assess the effect of abrogation of Wnt signaling. CD34^+^-derived ECs were seeded in a transwell insert coated with Matrigel at a density of 80,000 cells and cultured in medium supplemented with XAV 939 (0.1 and 1 µM). In the bottom of the transwell was seeded 45,000 bovine pericytes. After 4 days of coculture, the paracellular permeability and cell organization were evaluated. (D) Fluorescence microscopy images showing the expression of ZO-1 in untreated ECs or ECs treated with XAV 939 (1 µM) for 4 days. Scale bar corresponds to 50 µm. (E) Paracellular permeability of untreated ECs or ECs treated with 0.1 or 1 µM XAV939 for 4 days. Results are Mean ± SEM (*n* = 4).

To determine whether the activation of Wnt is required for the induction of barrier properties in CD34^+^-derived ECs, we cultured these cells alone up to 5 days and then exposed them to Wnt ligands/agonists. ECs respond rapidly to BIO, a specific pharmacological inhibitor of glycogen synthase kinase-3 (GSK-3) and thus an activator of Wnt signaling, or Wnt3a by increasing the expression of active β-catenin (**Fig. 5A**). The paracellular permeability of Wnt3a-treated ECs to Lucifer Yellow was statistical lower (*P*<0.01, *n* = 4) for short-term (5 days of monoculture + 1 day of Wnt3a treatment) and long-term (1 day of monoculture + 5 day of Wnt3a treatment) as compared to untreated cells (**Figs. 5A and 5B**). The effect of Wnt7a and BIO was only observed after 5-day treatment (**Figs. 5A and 5C**). During the induction process by Wnt3a or BIO, there is an increase in the expression and nuclear localization of total β-catenin (**Fig. 5D**), the up-regulation of β-catenin-associated transcription factor LEF1 gene (**Figs. 6A and 6B**), the up-regulation of claudin-1 gene expression (**Figs. 6A and 6B**) and the localization of claudin-1 at the cell-cell contacts (**Fig. 5D**). The localization of claudin-1 at the periphery of the cells might explain the restrictive permeability of ECs in co-culture with pericytes. To further confirm the role of Wnt pathway in the induction of BBB properties, we abrogated the Wnt signaling in ECs co-cultured with pericytes. ECs were seeded in a transwell insert coated with Matrigel while pericytes were seeded in the bottom of the transwell (**Figs. 6C–6E**). ECs were treated with the Wnt antagonist XAV-939 for 4 days by adding the inhibitor in the luminal side of the insert. The abrogation of Wnt pathway, in conditions that did not affect cell viability, increased the paracellular permeability of the EC monolayer to lucifer yellow. Overall, our gain and loss function experiments indicate that Wnt signaling is required for the BBB properties in CD34^+^-derived ECs co-cultured with pericytes.

## Discussion

Most *in vitro* BBB models currently available and fully characterized are based on primary BECs or cell lines from animal origin (bovine, porcine and murine) [12]. Only the hCMEC/D3 cell line, which retains morphological and functional characteristics of brain endothelium, is widely used as a human *in vitro* BBB model. Because this model has limitations, specifically high permeability to small hydrophilic compounds, there is a real need to develop new models of human BBB for performing reliable pharmacological and toxicological tests [5]. Here, we report the generation of a human *in vitro* BBB model from ECs derived from cord blood hematopoietic stem cells that is highly reproducible and stable for at least 20 days after its derivation. This model fills all the recommendations of the ECVAM workshop no 49 on the definition on an *in vitro* model [24]. Due to the relatively easy access to cord blood stem cells, this model can be adopted by the research community to study molecular mechanisms at the level of brain ECs in pathologies implicating the BBB such as neurodegenerative disorders (for example, Alzheimer’s disease and multiple sclerosis), stroke and traumatic brain injury, infectious processes and inflammatory pain.

To generate the human BBB model, we have used biological principles observed in the repair of BBB in the human body. The *in vivo* repair of the endothelium is mediated by endothelial progenitor cells (EPCs, characterized by the expression of CD34 marker among other markers [25]). The EPCs migrate to the sites of endothelial injury, incorporate in the endothelium and differentiate into ECs [26], [27]. Experimental results show that EPCs participate in neovascularization processes in the adult brain of mice after ischemia [28]. Therefore, our *in vitro* BBB model uses the biologic principles that exist in the human body.

Recently, a human *in vitro* BBB model from iPSCs has been reported [4], [29]. The model was based in the co-culture of iPSC-derived ECs with astrocytes. The derivation of ECs with BBB properties from pluripotent stem cells is suited for the large-scale production of BBB models, but suffers from several limitations including the complexity of the differentiation process, the reproducibility of the system and the uncertainty in terms of stability. Regarding this last point, the TEER of the monolayer reaches a peak after 25 h in culture but declines after 50 h. The use of retinoic acid substantially enhanced the BBB properties [29] (TEER up to 5000 Ω×cm^2^); however, the metabolic and the phenotypic characteristics of the BBB model were unclear. Furthermore, iPSCs are genetically reprogrammed and epigenetic modifications, which persist after reprogramming, could impact the application of these cells in basic research and drug development [30]. Finally, no correlation between the *in vitro* data and human permeability data was reported in this study.

During the submission of the present work, another study has reported a human *in vitro* model based on the co-culture of human endothelial cells derived from cord blood endothelial progenitor cells with astrocytes [5]. The study has demonstrated an up-regulation of tight junctions including occludin, the glucose transporter GLUT-1 and the active efflux transporter P-gp. However, the TEER value (60 Ω/cm^2^ versus 175 Ω/cm^2^) was lower and the permeability values to Lucifer yellow (1.23×10^−3 ^cm/min versus 0.61×10^−3 ^cm/min) were higher than our *in vitro* model [5]. Indeed, the paracellular permeability results were similar to those obtained with the hCMEC/D3 cell line. Furthermore, the monolayer presented optimal paracellular permeability during 4 days (between days 10 and 14) in opposition to our model that kept the paracellular permeability for at least 16 days (from day 4 up to day 20). In addition, the transporter activity of the cells for different ligands and correlation with human permeability data were unclear.

Our results also indicate that pericytes have superior BBB inductive properties on ECs derived from cord blood endothelial progenitor cells than astrocytes. This hypothesis is supported by our permeability results obtained with astrocytes (**Fig. S2A**). Further studies should be performed to know whether the differences observed in both systems are due to differences in the secretion of Wnt proteins by pericytes/astrocytes.

Our study is the first one showing a correlation between an *in vitro* human model prepared from stem cells and *in vivo* human data. Previous studies have demonstrated a correlation between human models and *in vivo* rat but not human data [2], [4]. Although further analysis should be performed with a larger library of compounds, the agreement (r^2^ = 0.89) between *in vitro* K_p,uu,brain_ and K_p,uu,CSF_ in humans is promising and suggests that the *in vitro* method developed here might be useful for identifying compounds susceptible to attain a desirable unbound drug concentrations in the human brain in drug discovery programs.

Our work provides *in vitro* evidence for a role of pericytes in the induction of BBB formation through the Wnt/β-catenin pathway. Although recent studies have shown that pericytes regulate the BBB, the underline mechanism was unclear. Previous studies have shown that angiopoietin-1 [31] and transforming growth factor-β [32] secreted by pericytes regulated the tight-junctions and the functionality of the BBB, respectively. In this work, we demonstrate that the BBB inductive properties of pericytes in ECs are mediated, at least in part, by Wnt/β-catenin signaling. This is in agreement with the fact that Wnt/β-catenin signaling regulates the induction and maintenance of BBB characteristics [13], [21]. Overall, our results contribute for a better understanding of the human BBB specifically for its induction and maintenance, while demonstrates the usefulness of our model for drug discovery programs.

## Supporting Information

Figure S1 Differentiation of human umbilical cord CD34^+^ cells into ECs and evaluation of their paracellular permeability. (A) Schematic representation of the differentiation of hematopoietic stem cells (CD34^+^CD45^+^CD31^+^KDR^−^vWF^−^CD14^−^) into ECs (2–3 weeks of differentiation) and evaluation of their paracellular permeability (Pe) using a Transwell system. (B) ECs immediately after differentiation (before culture in the Transwell system) express typical EC markers including CD31, VE-cadherin (VECAD), vWF and are able to incorporate AcLDL. Bar corresponds to 40 µm. (C) ECs after culture in the Transwell system have typical cobblestone morphology, express vWF and markers associated to hBECs such as claudin-5, ZO-1, and occludin; however, the expression of all these markers is discontinuous and cells do not express claudin-1 at cell-cell contacts. Bar corresponds to 50 µm. (D) Paracellular permeability of human ECs in monoculture and bovine ECs in co-culture with astrocytes for 12 days.
(TIF)

Figure S2 (A) Paracellular permeability of CD34^+^-derived ECs after co-culture with different types of cells in EGM-2 supplemented with 2% fetal calf serum (FCS). Results are Mean ± SEM (n = 6). (B) Characterization of bovine pericytes by phase contrast and Immunocytochemistry for the expression of vimentin, neuro-glial 2 (NG2), platelet-derived growth factor receptor beta (PDGFR-β), and α-smooth muscle actin (α-SMA). Scale bar corresponds to 50 µm. (C) The induction of BBB properties on CD34^+^-derived ECs requires the presence of pericytes in the co-culture system since pericyte-conditioned medium does not have the same BBB-inductive properties. CM stands for conditioned media.
(TIF)

Figure S3 (A) Double immunostaining for anti-human receptor for advanced glycation endproducts (RAGE) and anti-human organic cation/carnitine transporter (OCTN2; also known as SLC22A5) in monoculture (A.1) or in co-culture of CD34^+^-derived ECs with pericytes (A.2) at day 6. In the co-culture system, RAGE is present essentially in the luminal side of endothelial cells and OCTN2 in the abluminal side, while in monoculture, both markers seem to be located in the same plane. Bar corresponds to 10 µm. (B) Stability of the BBB properties after removal of the pericytes. CD34^+^-derived ECs were in co-culture with pericytes for 14 days (1) or in co-culture for 6 days and then 8 days in monoculture (2).
(TIF)

Table S1 Antibodies used for immunofluorescence^⧫^, flow cytometry^★^ and Western blot^▪^.
(DOC)

Table S2 Primers used for quantitative real time-PCR and non-quantitative PCR*.
(DOC)

Table S3 Down-regulated genes in the microarray. Gene expression on CD34^+^-derived ECs in co-culture at day 6 and 3 was significantly different regarding BBB markers, specifically for efflux transporters including solute carrier family members SLC2A3, SLC6A6 and SLC47A1 (downregulated at day 6) and non-BBB markers such as channels and extracellular matrix. These results show that the induction process is a dynamic process affecting the expression of transporters, channels and ECM components.
(DOC)

Table S4 Up-regulated genes in the microarray. Gene expression on CD34^+^-derived ECs in co-culture at day 6 and 3 was significantly different regarding BBB markers, specifically for efflux transporters including solute carrier family members SLC30A3, SLC26A10, SLC13A3 and SLC44A5 (upregulated at day 6) and non-BBB markers such as channels and extracellular matrix.
(DOC)

Text S1 Materials and Methods.
(DOC)
