# Supplementary figures and images for: A Stable and Reproducible Human Blood-Brain Barrier Model Derived from Hematopoietic Stem Cells
Source: PLoS One. 2014 Jun 17;9(6):e99733. doi: 10.1371/journal.pone.0099733 (PMC4061029; doi:10.1371/journal.pone.0099733)

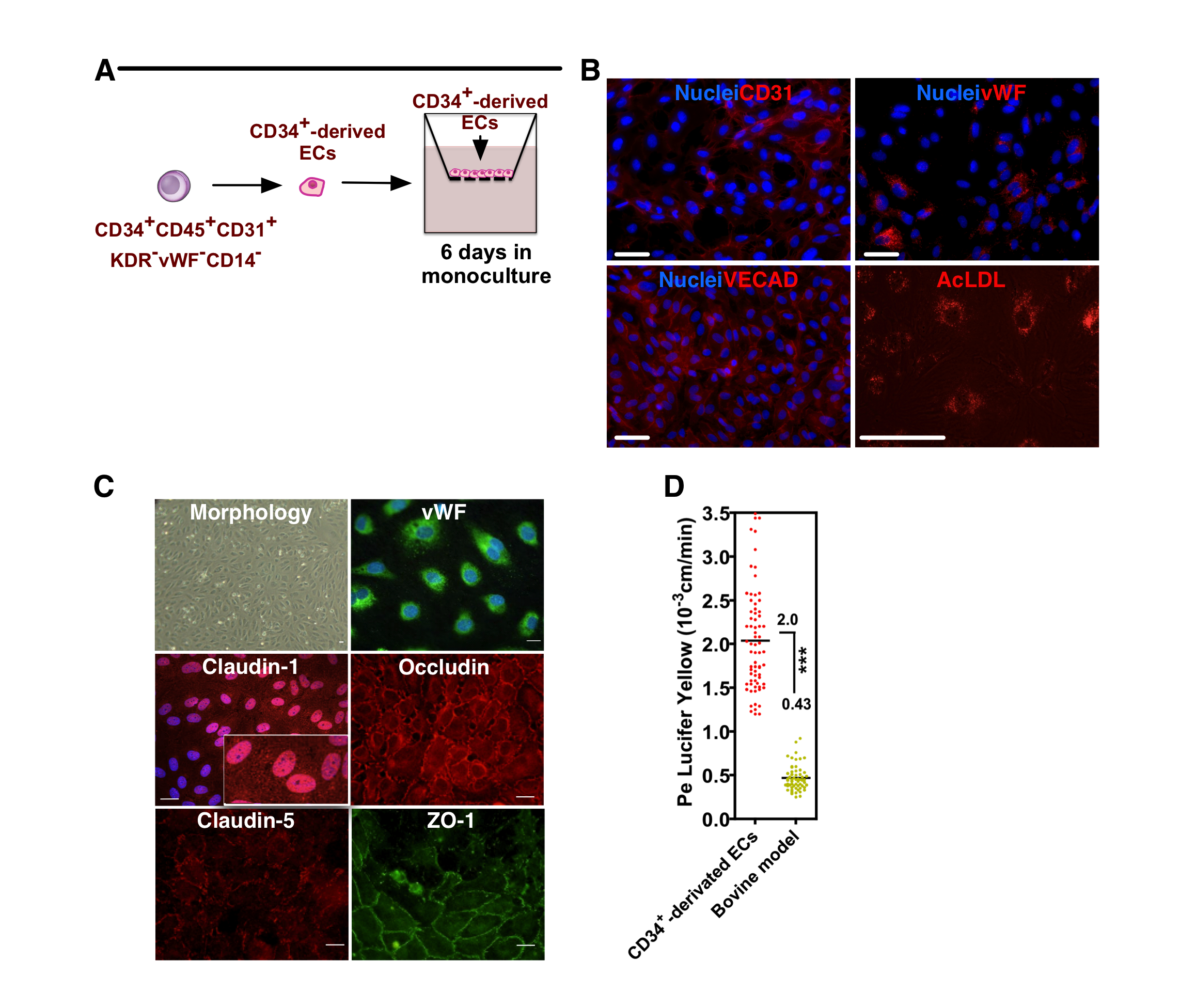

Supplement: Figure S1 — Differentiation of human umbilical cord CD34+ cells into ECs and evaluation of their paracellular permeability. (A) Schematic representation of the differentiation of hematopoietic stem cells (CD34+CD45+CD31+KDR−vWF−CD14−) into ECs (2–3 weeks of differentiation) and evaluation of their paracellular permeability (Pe) using a Transwell system. (B) ECs immediately after differentiation (before culture in the Transwell system) express typical EC markers including CD31, VE-cadherin (VECAD), vWF and are able to incorporate AcLDL. Bar corresponds to 40 µm. (C) ECs after culture in the Transwell system have typical cobblestone morphology, express vWF and markers associated to hBECs such as claudin-5, ZO-1, and occludin; however, the expression of all these markers is discontinuous and cells do not express claudin-1 at cell-cell contacts. Bar corresponds to 50 µm. (D) Paracellular permeability of human ECs in monoculture and bovine ECs in co-culture with astrocytes for 12 days. (TIF) [file pone.0099733.s001.tif]

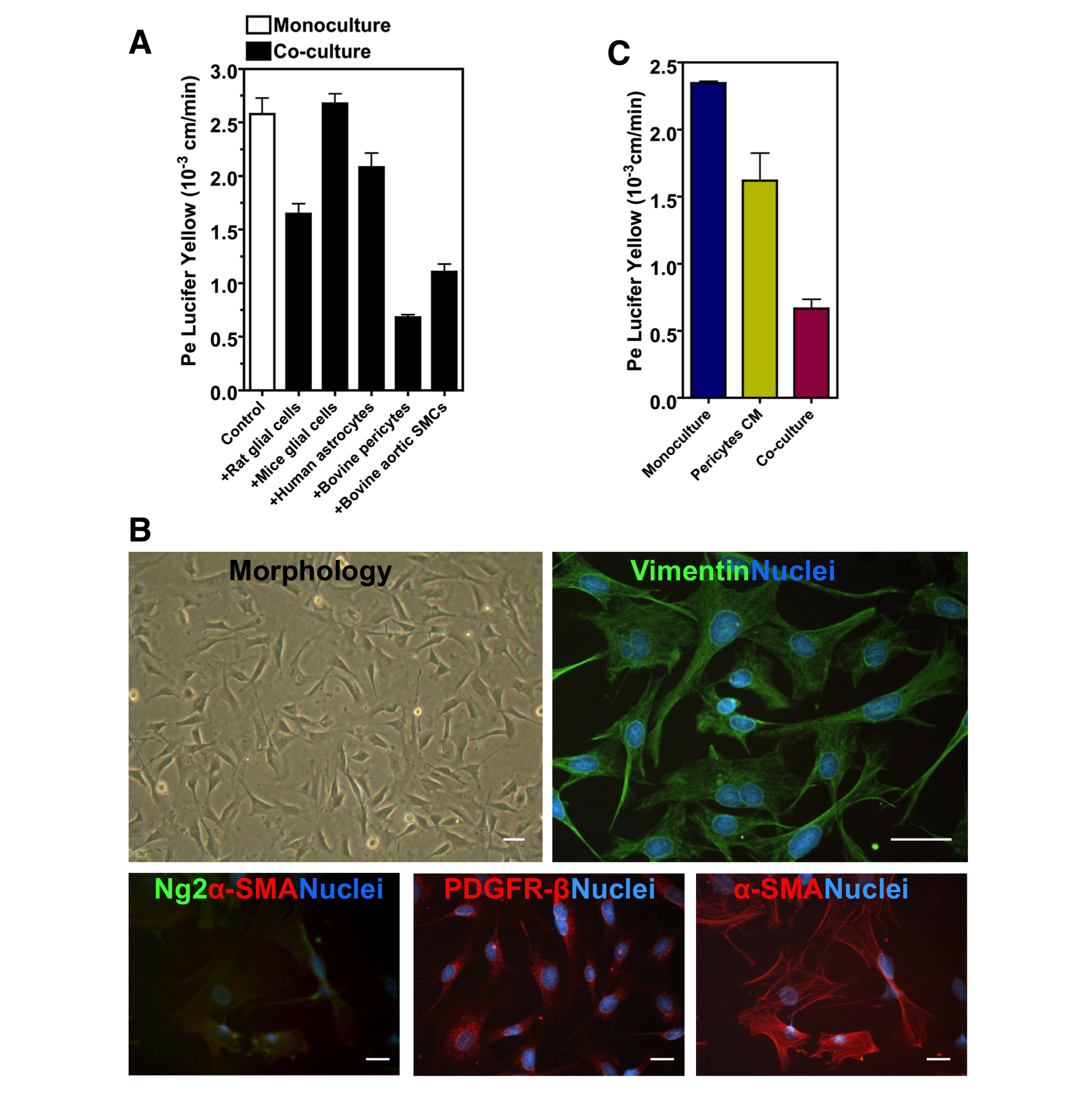

Supplement: Figure S2 — (A) Paracellular permeability of CD34+-derived ECs after co-culture with different types of cells in EGM-2 supplemented with 2% fetal calf serum (FCS). Results are Mean ± SEM (n = 6). (B) Characterization of bovine pericytes by phase contrast and Immunocytochemistry for the expression of vimentin, neuro-glial 2 (NG2), platelet-derived growth factor receptor beta (PDGFR-β), and α-smooth muscle actin (α-SMA). Scale bar corresponds to 50 µm. (C) The induction of BBB properties on CD34+-derived ECs requires the presence of pericytes in the co-culture system since pericyte-conditioned medium does not have the same BBB-inductive properties. CM stands for conditioned media. (TIF) [file pone.0099733.s002.tif]

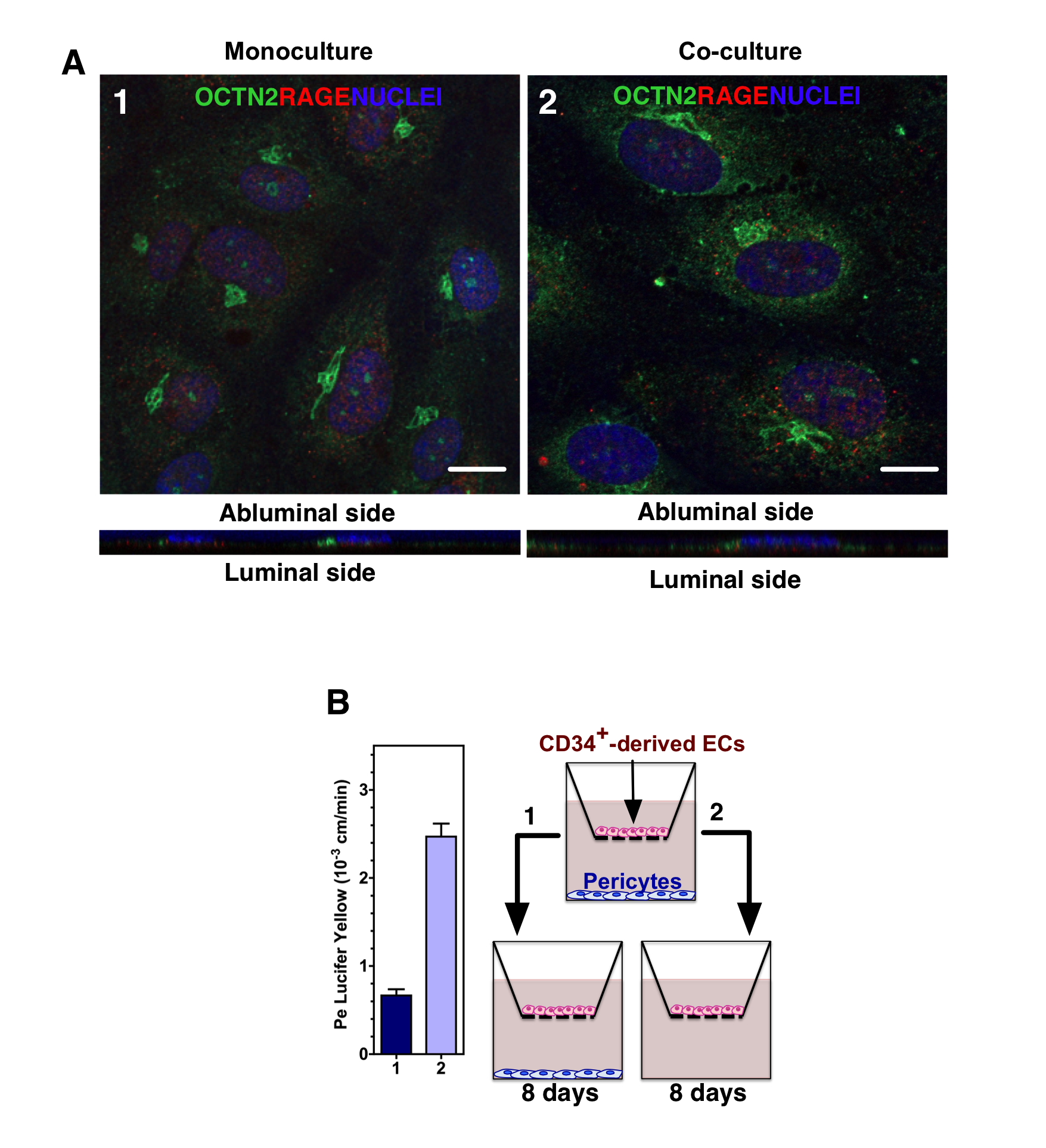

Supplement: Figure S3 — (A) Double immunostaining for anti-human receptor for advanced glycation endproducts (RAGE) and anti-human organic cation/carnitine transporter (OCTN2; also known as SLC22A5) in monoculture (A.1) or in co-culture of CD34+-derived ECs with pericytes (A.2) at day 6. In the co-culture system, RAGE is present essentially in the luminal side of endothelial cells and OCTN2 in the abluminal side, while in monoculture, both markers seem to be located in the same plane. Bar corresponds to 10 µm. (B) Stability of the BBB properties after removal of the pericytes. CD34+-derived ECs were in co-culture with pericytes for 14 days (1) or in co-culture for 6 days and then 8 days in monoculture (2). (TIF) [file pone.0099733.s003.tif]
