# Supplementary material for: A Stable and Reproducible Human Blood-Brain Barrier Model Derived from Hematopoietic Stem Cells
Source: PLoS One. 2014 Jun 17;9(6):e99733. doi: 10.1371/journal.pone.0099733 (PMC4061029; doi:10.1371/journal.pone.0099733)
Supplement: Table S1 — Antibodies used for immunofluorescence⧫, flow cytometry★ and Western blot▪. (DOC) [file pone.0099733.s004.doc]

**Table S1**- Antibodies used for immunofluorescence^^, flow cytometry^★^ and Western blot^^.

|  | **Antibody** | **Dilution** | **Reference** | | **Supplier** | **Fixation** |
| --- | --- | --- | --- | --- | --- | --- |
| **Endothelial cells** | Rabbit anti-occludin | 1/200^◆^ | 71-500 | | Life Technologies | 4% PFA |
|  | Rabbit anti-ZO1 | 1/200^◆^ | 61-7300 | | Life Technologies | 4% PFA |
|  | Rabbit anti-claudin5 | 1/100^◆^ | 34-1600 | | Life Technologies | Methanol/acetone |
|  | Rabbit anti-claudin1,3 | 1/10^◆^ | 71-7800 | | Life Technologies | 4% PFA |
|  | Rabbit anti-claudin1 | 1/25^◆^ | Ab15098 | | Abcam | 4% PFA^◆^ |
|  | Mouse anti-JAM1 | 1/100^◆^ | 552147 | | Becton Dickinson | Methanol/acetone |
|  | Mouse anti-Pgp | 1/10^◆^ | GTX23364 | | GeneTex | 4% PFA |
|  | Goat anti-RAGE | 1/100^◆^ | Sc-8230 | | Santa Cruz Biotechnology | 4% PFA |
|  | Rabbit anti-Wnt3 | 1/500^◼^ | Sc-28824 | | Santa Cruz Biotechnology | N/A |
|  | Goat anti-Wnt7A | 1/250^◼^ | Sc-26361 | | Santa Cruz Biotechnology | N/A |
|  | Goat anti-Shh | 1/250^◼^ | Sc-1194 | | Santa Cruz Biotechnology | N/A |
|  | Rabbit anti-AHNAK | 1/50^◆^ | Sc-98373 | | Santa Cruz Biotechnology | 4% PFA |
|  | Mouse anti-PECAM1 | 1/50^★,◆^ | M0823 | | DAKO | 4% PFA |
|  | Mouse anti-VE-cadherin | 1/50^◆^ | Sc-9989 | | Santa Cruz Biotechnology | 4% PFA |
|  | Mouse anti-von Willebrand Factor | 1/50^◆^ | M0616 | | DAKO | 4% PFA |
|  | FITC mouse anti-CD106 (VCAM-1) | 1/50^★^ | 551146 | | BD Biosciences | N/A |
|  | Mouse anti-CD40 | 1/50^★^ | Sc-65264 | | Santa Cruz Biotechnology | N/A |
|  | Mouse anti-ICAM1 | 1/50^★^ | Sc-107 | | Santa Cruz Biotechnology | N/A |
|  | Mouse anti-ICAM2 | 1/50^★^ | Sc-23935 | | Santa Cruz Biotechnology | N/A |
|  | Mouse anti-active beta catenin | 1/300^★^ | 05-665 | | Millipore | 4% PFA |
|  | Rabbit anti-total beta catenin | 1/2000^◆^, 1/4000^◼^ | Ab6302 | | Abcam | 4% PFA |
|  | Rabbit Anti-OCTN2 | 1/50^◆^ | Home-made antibody | It was kindly supplied by Dr Nałecz KA, Nencki Institute of Experimental Biology, Warsaw, Poland. | | 4% PFA |
|  | Goat Anti-RAGE | 1/100^◆^ | Sc-8230 | Santa Cruz Biotechnology | | 4% PFA |
|  | Mouse anti- alpha tubulin | 1/1000^◼^ | T6199 | Sigma | | N/A |
| **Pericytes** | Rabbit anti-PDGFR-beta | 1/100^◆^ | Ab51092 | Abcam | | 4% PFA |
|  | Rabbit anti-alpha SMA | 1/200^◆^ | M0851 | DAKO | | 4% PFA |
|  | Rabbit anti-NG2 | 1/200^◆^ | Ab5320 | Millipore | | 4% PFA |
| **Secondary antibodies** | Alexa Fluor 488 anti rabbit | 1/200^◆^ | A11034 | Molecular Probes | | 4% PFA |
|  | Alexa Fluor 568 anti rabbit | 1/200^◆^ | A11036 | Molecular Probes | | 4% PFA |
|  | Alexa Fluor 568 anti mouse | 1/200^◆^ | A11031 | Molecular Probes | | 4% PFA |
|  | Alexa Fluor 568 anti goat | 1/200^◆^ | A11057 | Molecular Probes | | 4% PFA |
|  | Cy3 anti mouse | 1/100^◆^ | C2181 | Sigma | | 4% PFA |
|  | Phycoerythrin anti rabbit | 1/20^★^ | F0110 | R&D Systems | | 4% PFA, Methanol |
|  | Cy3 anti rabbit | 1/100^◆^ | 111-165-144 | Jackson Immunoresearch | | 4% PFA |
|  | Phycoerythrin anti mouse | 1/100^★^ | Sc-358926 | Santa Cruz Biotechnology | | N/A |
|  | Alkaline phosphatase anti mouse | 1/5000^◼^ | RPN5781 | GE Healthcare | | N/A |
|  | Alkaline phosphatase anti rabbit | 1/5000^◼^ | RPN5783 | GE Healthcare | | N/A |
|  | Alkaline phosphatase anti goat | 1/3000^◼^ | 705-055-003 | Jackson Immunoresearch | | N/A |
| **Other reagents** | Hoechst 33258 | 4 mg/mL^◆^ | 190304 | ICN | | 4% PFA |
|  | DAPI | 2 μg/mL^◆^ | D9542 | Sigma | | 4% PFA |
