# Supplementary material for: A Stable and Reproducible Human Blood-Brain Barrier Model Derived from Hematopoietic Stem Cells
Source: PLoS One. 2014 Jun 17;9(6):e99733. doi: 10.1371/journal.pone.0099733 (PMC4061029; doi:10.1371/journal.pone.0099733)
Supplement: Table S2 — Primers used for quantitative real time-PCR and non-quantitative PCR*. (DOC) [file pone.0099733.s005.doc]

**Table S2**- Primers used for quantitative real time-PCR and non-quantitative PCR*.

| **Gene** | **Forward sequence** | **Reverse sequence** |
| --- | --- | --- |
| GAPDH | AGCCACATCGCTCAGACACC | GTACTCAGCGCCAGCATCG |
| CLDN-1 | GAAAGACTACGTGTGACA | GGTCCTAATGTTAATGATAGTATC |
| CLDN-3 | ATCACGTCGCAGAACATC | TACACCTTGCACTGCATCTG |
| CLDN-5 | TTAACAGACGGAATGAAGTT | AAGCGAAATCCTCAGTCT |
| OCLDN | TTCTGGATCTCTATATGGTTCA | CCACAACACAGTAGTGATAC |
| ZO-1 | CCTGAACCAGTATCTGATAA | AATCTTCTCACTCCTTCTG |
| SLC6A8 | TGAGAGAATGAGATTTCTGCTTGT | TAGGGCTCACAGGGATGG |
| SLC3A2 | TTGGCTCCAAGGAAGATT | GAGTAAGGTCCAGAATGACA |
| SLC2A1 | GAGACACTTGCCTTCTTC | GCTTTGTAGTTCATAGTTCG |
| SLC7A5 | TTGACACCACTAAGATGAT | GTAGCAATGAGGTTCCAA |
| SLC7A1 | CCTCCTGAGACATCTTTG | CTGGAATATGACGGGAAG |
| SLC16A1 | ACACAAAGCCAATAAGAC | ACAGAATCCAACATAGGTA |
| TFRC | ATGCTGACAATAACACAA | CCAAGTAGCCAATCATAA |
| WNT3A | ATCCTCTGCCTCAAATTCT | TTCGTCTAACTCCGTTGG |
| WNT7A | CGGGAGATCAAGCAGAATG | CGTGGCACTTACATTCCAG |
| WNT7B | GCTTCGTCAAGTGCAACA | GGAGTGGATGTGCAAAATG |
| FZD4 | TACCTCACAAAACCCCCATCC | GGCTGTATAAGCCAGCATCAT |
| FZD6 | TCGTCAGTACCATATCCCATG | CCCATTCTGTGCATGTCTTTT |
| FZD7 | GATGATAACGGCGATGTGA | AACAAAGCAGCCACCGCAGAC |
| APCDD1 | GGAGTCACAGTGCCATCACAT | CCTGACCTTACTTCACAGCCT |
| LEF1 | AAGGAACACTGACATCAATT | TTTGGAACTTGGCTCTTG |
| P-GP* | GCCTGGCAGCTGGAAGACAAATACACAAAATT | CAGACAGCAGCTGACAGTCCAAGAACAGGACT |
| BCRP* | TGGCTGTCATGGCTTCAGTA | GCCACGTGATTCTTCCACAA |
| MRP1* | ACCAAGACGTATCAGGTGGCC | CTGTCTGGGCATCCAGGAT |
| MRP2* | CCAATCTACTCTCACTTCAGCGAGA | AGATCCAGCTCAGGTCGGTACC |
| MRP4* | AAGTGAACAACCTCCAGTTCCA | CCGGAGCTTTCAGAATTGAC |
| MRP5* | AGTGGCACTGTCAGATCAAATT | TTGTTCTCTGCAGCAGCAAAC |
| hTRF* | CTGCTATGGGACTATTGCTGTG | CCGACAACTTTCTCTTCAGGTC |
| RAGE* | CTCGAATGGAAACTGAACAC | CTGGTAGTTAGACTTGGTCTC |
| LRP1* | GCATCCTGATCGAGCACCTG | GCCAATGAGGTAGCTGGTGG |
