# Supplementary material for: A Stable and Reproducible Human Blood-Brain Barrier Model Derived from Hematopoietic Stem Cells
Source: PLoS One. 2014 Jun 17;9(6):e99733. doi: 10.1371/journal.pone.0099733 (PMC4061029; doi:10.1371/journal.pone.0099733)
Supplement: Table S3 — Down-regulated genes in the microarray. Gene expression on CD34+-derived ECs in co-culture at day 6 and 3 was significantly different regarding BBB markers, specifically for efflux transporters including solute carrier family members SLC2A3, SLC6A6 and SLC47A1 (downregulated at day 6) and non-BBB markers such as channels and extracellular matrix. These results show that the induction process is a dynamic process affecting the expression of transporters, channels and ECM components. (DOC) [file pone.0099733.s006.doc]

**Table S3-** Down-regulated genes in the microarray. Gene expression on CD34^+^-derived ECs in co-culture at day 6 and 3 was significantly different regarding BBB markers, specifically for efflux transporters including solute carrier family members SLC2A3, SLC6A6 and SLC47A1 (downregulated at day 6) and non-BBB markers such as channels and extracellular matrix. These results show that the induction process is a dynamic process affecting the expression of transporters, channels and ECM components.

|  |  |  | Co-culture 6 days versus Mono-culture 6 days |  |
| --- | --- | --- | --- | --- |
| Unique ID | Target ID | Gene Symbol | Gene Name | M Value |
| A_23_P328740 | BC012317 | LINCR | likely ortholog of mouse lung-inducible Neutralized-related C3HC4 RING domain protein | -2.38 |
| A_24_P659122 | AK125790 | LOC401357 | hypothetical LOC401357 | -2.38 |
|  |  |  |  |  |
|  |  |  | Co-culture 6 days versus Co-culture 3 days |  |
| Unique ID | Target ID | Gene Symbol | Gene Name | M Value |
| A_23_P259314 | NM_001008 | RPS4Y1 | ribosomal protein S4, Y-linked 1" | -12.62 |
| A_23_P324384 | NM_001039567 | RPS4Y2 | ribosomal protein S4, Y-linked 2 | -11.61 |
| A_23_P254944 | NM_000853 | GSTT1 | glutathione S-transferase theta 1 | -9.39 |
| A_23_P217797 | AF000984 | DDX3Y | DEAD (Asp-Glu-Ala-Asp) box polypeptide 3, Y-linked" | -8.83 |
| A_23_P73848 | NR_001544 | CYorf14 | chromosome Y open reading frame 14 | -6.89 |
| A_24_P325205 | NM_003471 | KCNAB1 | potassium voltage-gated channel, shaker-related subfamily, beta member 1" | -6.67 |
| A_23_P364792 | NM_001005852 | CYorf15A | chromosome Y open reading frame 15A | -6.57 |
| A_24_P237511 | NM_004681 | EIF1AY | eukaryotic translation initiation factor 1A, Y-linked" | -6.55 |
| A_23_P121441 | NM_014893 | NLGN4Y | neuroligin 4, Y-linked" | -6.50 |
| A_23_P152002 | NM_004049 | BCL2A1 | BCL2-related protein A1 | -6.24 |
| A_23_P113613 | NM_022842 | CDCP1 | CUB domain containing protein 1 | -6.08 |
| A_23_P44494 | NM_003471 | KCNAB1 | potassium voltage-gated channel, shaker-related subfamily, beta member 1" | -6.03 |
| A_23_P149345 | NM_015967 | PTPN22 | protein tyrosine phosphatase, non-receptor type 22 (lymphoid)" | -5.82 |
| A_24_P319001 | NM_000853 | GSTT1 | glutathione S-transferase theta 1 | -5.71 |
| A_24_P182929 | NM_003471 | KCNAB1 | potassium voltage-gated channel, shaker-related subfamily, beta member 1" | -5.68 |
| A_23_P33903 | NM_014893 | NLGN4Y | neuroligin 4, Y-linked" | -5.47 |
| A_24_P942743 | NM_003411 | ZFY | zinc finger protein, Y-linked | -5.38 |
| A_23_P139881 | NM_001759 | CCND2 | cyclin D2 | -5.32 |
| A_23_P150457 | NM_006691 | LYVE1 | lymphatic vessel endothelial hyaluronan receptor 1 | -5.11 |
| A_23_P400449 | NM_020927 | VAT1L | vesicle amine transport protein 1 homolog (T. californica)-like | -5.02 |
| A_24_P306443 | NM_001033515 | LOC100132288 | hypothetical protein LOC100132288 | -5.01 |
| A_23_P383009 | NM_000599 | IGFBP5 | insulin-like growth factor binding protein 5 | -4.99 |
| A_23_P80570 | NM_001086 | AADAC | arylacetamide deacetylase (esterase) | -4.87 |
| A_23_P138524 | NM_198148 | CPXM2 | carboxypeptidase X (M14 family), member 2" | -4.85 |
| A_23_P56505 | NM_000885 | ITGA4 | integrin, alpha 4 (antigen CD49D, alpha 4 subunit of VLA-4 receptor)" | -4.69 |
| A_32_P231179 | NM_144705 | TEKT4 | tektin 4 | -4.66 |
| A_23_P96658 | ENST00000382832 | CYorf15B | chromosome Y open reading frame 15B | -4.64 |
| A_23_P66798 | NM_002276 | KRT19 | keratin 19 | -4.52 |
| A_24_P160401 | NM_178181 | CDCP1 | CUB domain containing protein 1 | -4.48 |
| A_24_P216625 | NR_001544 | CYorf14 | chromosome Y open reading frame 14 | -4.44 |
| A_23_P314755 | NM_003155 | STC1 | stanniocalcin 1 | -4.33 |
| A_23_P1682 | NM_138788 | TMEM45B | transmembrane protein 45B | -4.28 |
| A_24_P49260 | NM_018327 | SPTLC3 | serine palmitoyltransferase, long chain base subunit 3 | -4.23 |
| A_23_P74609 | NM_015714 | G0S2 | G0/G1switch 2 | -4.17 |
| A_23_P89871 | NM_018355 | ZNF415 | zinc finger protein 415 | -4.14 |
| A_32_P224302 | NM_003436 | ZNF135 | zinc finger protein 135 | -4.08 |
| A_32_P94199 | BC068588 | LOC653071 | similar to CG32820-PA, isoform A | -4.02 |
| A_24_P307993 | BC035312 | CYorf15B | chromosome Y open reading frame 15B | -3.97 |
| A_23_P121987 | NM_033035 | TSLP | thymic stromal lymphopoietin | -3.94 |
| A_23_P201181 | NM_012411 | PTPN22 | protein tyrosine phosphatase, non-receptor type 22 (lymphoid)" | -3.90 |
| A_32_P55840 | ENST00000377186 | LOC730405 | hypothetical protein LOC730405 | -3.86 |
| A_24_P245379 | NM_002575 | SERPINB2 | serpin peptidase inhibitor, clade B (ovalbumin), member 2" | -3.85 |
| A_23_P4953 | NM_018215 | PNMAL1 | PNMA-like 1 | -3.81 |
| A_23_P421664 | NM_006366 | CAP2 | CAP, adenylate cyclase-associated protein, 2 (yeast)" | -3.71 |
| A_23_P419714 | NM_001018072 | BTBD11 | BTB (POZ) domain containing 11 | -3.68 |
| A_23_P329835 | NM_007125 | UTY | ubiquitously transcribed tetratricopeptide repeat gene, Y-linked" | -3.67 |
| A_24_P389415 | NM_007257 | PNMA2 | paraneoplastic antigen MA2 | -3.64 |
| A_23_P371039 | NM_002531 | NTSR1 | neurotensin receptor 1 (high affinity) | -3.59 |
| A_23_P361448 | NM_144665 | SESN3 | sestrin 3 | -3.59 |
| A_32_P34844 | NM_199355 | ADAMTS18 | ADAM metallopeptidase with thrombospondin type 1 motif, 18" | -3.57 |
| A_24_P296808 | NM_018215 | PNMAL1 | PNMA-like 1 | -3.53 |
| A_23_P422911 | NM_153456 | HS6ST3 | heparan sulfate 6-O-sulfotransferase 3 | -3.50 |
| A_32_P114003 | NR_024360 | LOC100192378 | hypothetical LOC100192378 | -3.50 |
| A_23_P166109 | NM_198391 | FLRT3 | fibronectin leucine rich transmembrane protein 3 | -3.46 |
| A_23_P348227 | NM_003436 | ZNF135 | zinc finger protein 135 | -3.45 |
| A_23_P350001 | NM_000855 | GUCY1A2 | guanylate cyclase 1, soluble, alpha 2" | -3.41 |
| A_23_P420863 | NM_022162 | NOD2 | nucleotide-binding oligomerization domain containing 2 | -3.40 |
| A_23_P353865 | AB041269 | KRT19P2 | keratin 19 pseudogene 2 | -3.40 |
| A_23_P64539 | NM_000559 | HBG1 | hemoglobin, gamma A" | -3.36 |
| A_23_P97402 | NM_020439 | CAMK1G | calcium/calmodulin-dependent protein kinase IG | -3.34 |
| A_23_P15004 | NM_199355 | ADAMTS18 | ADAM metallopeptidase with thrombospondin type 1 motif, 18" | -3.34 |
| A_23_P117104 | NM_001651 | AQP5 | aquaporin 5 | -3.32 |
| A_23_P137238 | NM_004653 | JARID1D | jumonji, AT rich interactive domain 1D" | -3.27 |
| A_32_P80245 | NM_001109809 | ZFP57 | zinc finger protein 57 homolog (mouse) | -3.26 |
| A_23_P395438 | NM_053044 | HTRA3 | HtrA serine peptidase 3 | -3.23 |
| A_23_P217379 | NM_033641 | COL4A6 | collagen, type IV, alpha 6" | -3.21 |
| A_32_P181222 | NM_002247 | KCNMA1 | potassium large conductance calcium-activated channel, subfamily M, alpha member 1" | -3.16 |
| A_23_P53137 | NM_000559 | HBG1 | hemoglobin, gamma A" | -3.16 |
| A_23_P154037 | NM_001159 | AOX1 | aldehyde oxidase 1 | -3.16 |
| A_23_P112698 | NM_007257 | PNMA2 | paraneoplastic antigen MA2 | -3.15 |
| A_23_P49376 | NM_000078 | CETP | cholesteryl ester transfer protein, plasma" | -3.11 |
| A_23_P378555 | NM_152615 | PARP15 | poly (ADP-ribose) polymerase family, member 15 | -3.09 |
| A_23_P160004 | NM_182660 | UTY | ubiquitously transcribed tetratricopeptide repeat gene, Y-linked" | -3.09 |
| A_23_P318881 | NM_170601 | SIAE | sialic acid acetylesterase | -3.07 |
| A_23_P112482 | NM_004925 | AQP3 | aquaporin 3 (Gill blood group) | -3.07 |
| A_23_P434398 | NM_153235 | TXLNB | taxilin beta | -3.02 |
| A_23_P48414 | NM_003914 | CCNA1 | cyclin A1 | -3.01 |
| A_23_P369899 | NM_015444 | TMEM158 | transmembrane protein 158 | -3.01 |
| A_24_P39919 | NM_023926 | ZSCAN18 | zinc finger and SCAN domain containing 18 | -3.00 |
| A_23_P69171 | NM_033050 | SUCNR1 | succinate receptor 1 | -3.00 |
| A_32_P100830 | NM_153209 | KIF19 | kinesin family member 19 | -3.00 |
| A_24_P917819 | DQ179139 | C21orf99 | chromosome 21 open reading frame 99 | -2.99 |
| A_24_P290709 | CR593166 | TOM1L1 | target of myb1 (chicken)-like 1 | -2.99 |
| A_23_P10542 | NM_053044 | HTRA3 | HtrA serine peptidase 3 | -2.96 |
| A_32_P215700 | NM_181643 | C1orf88 | chromosome 1 open reading frame 88 | -2.95 |
| A_24_P339429 | NM_021012 | KCNJ12 | potassium inwardly-rectifying channel, subfamily J, member 12" | -2.95 |
| A_23_P84063 | NM_016522 | NTM | neurotrimin | -2.94 |
| A_32_P83098 | NM_000336 | SCNN1B | sodium channel, nonvoltage-gated 1, beta" | -2.93 |
| A_23_P57658 | NM_020386 | HRASLS | HRAS-like suppressor | -2.91 |
| A_23_P114084 | NM_000444 | PHEX | phosphate regulating endopeptidase homolog, X-linked" | -2.89 |
| A_23_P39550 | NM_030923 | TMEM163 | transmembrane protein 163 | -2.88 |
| A_23_P153185 | NM_002575 | SERPINB2 | serpin peptidase inhibitor, clade B (ovalbumin), member 2" | -2.87 |
| A_23_P404016 | BC026362 | KIF19 | kinesin family member 19 | -2.87 |
| A_23_P136116 | NM_001004320 | TMEM195 | transmembrane protein 195 | -2.87 |
| A_32_P68103 | NM_012409 | PRND | prion protein 2 (dublet) | -2.86 |
| A_24_P66233 | NR_001543 | TTTY14 | testis-specific transcript, Y-linked 14" | -2.86 |
| A_32_P204795 | ENST00000299997 | LOC100128252 | similar to MGC9913 protein | -2.85 |
| A_23_P28948 | NM_014012 | REM1 | RAS (RAD and GEM)-like GTP-binding 1 | -2.85 |
| A_23_P324754 | NM_018689 | KIAA1199 | KIAA1199 | -2.84 |
| A_23_P144746 | NM_182594 | ZNF454 | zinc finger protein 454 | -2.84 |
| A_23_P106405 | NM_002487 | NDN | necdin homolog (mouse) | -2.83 |
| A_23_P118493 | NM_005486 | TOM1L1 | target of myb1 (chicken)-like 1 | -2.83 |
| A_23_P54100 | NM_001437 | ESR2 | estrogen receptor 2 (ER beta) | -2.83 |
| A_23_P361085 | NR_003038 | SNHG5 | small nucleolar RNA host gene 5 (non-protein coding) | -2.82 |
| A_23_P205164 | NM_006237 | POU4F1 | POU class 4 homeobox 1 | -2.82 |
| A_32_P225472 | XM_001125792 | LOC727834 | hypothetical LOC727834 | -2.77 |
| A_23_P371145 | NM_138430 | ADPRHL1 | ADP-ribosylhydrolase like 1 | -2.73 |
| A_23_P161439 | NM_006829 | C10orf116 | chromosome 10 open reading frame 116 | -2.71 |
| A_24_P357847 | BC030813 | IGK@ | immunoglobulin kappa locus | -2.71 |
| A_23_P397285 | NM_017527 | LY6K | lymphocyte antigen 6 complex, locus K" | -2.70 |
| A_24_P33982 | NM_001085423 | C17orf60 | chromosome 17 open reading frame 60 | -2.70 |
| A_23_P55682 | NM_023926 | ZSCAN18 | zinc finger and SCAN domain containing 18 | -2.69 |
| A_23_P150394 | NM_022003 | FXYD6 | FXYD domain containing ion transport regulator 6 | -2.69 |
| A_23_P17663 | NM_002462 | MX1 | myxovirus (influenza virus) resistance 1, interferon-inducible protein p78 (mouse)" | -2.69 |
| A_23_P142075 | NM_001611 | ACP5 | acid phosphatase 5, tartrate resistant" | -2.67 |
| A_32_P449517 | NM_001033515 | LOC100132288 | hypothetical protein LOC100132288 | -2.66 |
| A_24_P324883 | AK097143 | FLJ39824 | hypothetical LOC441173 | -2.66 |
| A_23_P127220 | NM_021800 | DNAJC12 | DnaJ (Hsp40) homolog, subfamily C, member 12" | -2.66 |
| A_23_P501010 | NM_000494 | COL17A1 | collagen, type XVII, alpha 1" | -2.65 |
| A_23_P150768 | NM_007256 | SLCO2B1 | solute carrier organic anion transporter family, member 2B1" | -2.65 |
| A_32_P70315 | NM_003256 | TIMP4 | TIMP metallopeptidase inhibitor 4 | -2.64 |
| A_23_P120125 | NM_199235 | COLEC11 | collectin sub-family member 11 | -2.60 |
| A_23_P8640 | NM_001039966 | GPER | G protein-coupled estrogen receptor 1 | -2.58 |
| A_23_P115726 | NM_194298 | SLC16A9 | solute carrier family 16, member 9 (monocarboxylic acid transporter 9)" | -2.57 |
| A_24_P48204 | NM_003004 | SECTM1 | secreted and transmembrane 1 | -2.57 |
| A_32_P107876 | NM_025074 | FRAS1 | Fraser syndrome 1 | -2.55 |
| A_32_P148118 | XM_001717196 | LOC642424 | similar to hCG1742442 | -2.54 |
| A_23_P357101 | NM_145298 | APOBEC3F | apolipoprotein B mRNA editing enzyme, catalytic polypeptide-like 3F" | -2.53 |
| A_23_P360754 | NM_005099 | ADAMTS4 | ADAM metallopeptidase with thrombospondin type 1 motif, 4" | -2.53 |
| A_24_P196658 | NM_005486 | TOM1L1 | target of myb1 (chicken)-like 1 | -2.51 |
| A_23_P38630 | NM_001050 | SSTR2 | somatostatin receptor 2 | -2.51 |
| A_23_P119886 | NM_001486 | GCKR | glucokinase (hexokinase 4) regulator | -2.49 |
| A_23_P120931 | NM_014508 | APOBEC3C | apolipoprotein B mRNA editing enzyme, catalytic polypeptide-like 3C | -2.49 |
| A_23_P212050 | NM_000055 | BCHE | butyrylcholinesterase | -2.49 |
| A_24_P339858 | NR_026547 | C21orf90 | chromosome 21 open reading frame 90 | -2.48 |
| A_23_P417261 | NM_144715 | EFHB | EF-hand domain family, member B" | -2.48 |
| A_24_P402242 | NM_000090 | COL3A1 | collagen, type III, alpha 1" | -2.46 |
| A_24_P323148 | NM_182573 | LYPD5 | LY6/PLAUR domain containing 5 | -2.43 |
| A_23_P60210 | NM_006911 | RLN1 | relaxin 1 | -2.41 |
| A_23_P43095 | NM_024721 | ZFHX4 | zinc finger homeobox 4 | -2.41 |
| A_23_P258612 | NM_016529 | ATP8A2 | ATPase, aminophospholipid transporter-like, class I, type 8A, member 2" | -2.40 |
| A_23_P397293 | NM_017527 | LY6K | lymphocyte antigen 6 complex, locus K" | -2.39 |
| A_23_P254816 | NM_004609 | TCF15 | transcription factor 15 (basic helix-loop-helix) | -2.37 |
| A_32_P225092 | NM_019590 | KIAA1217 | KIAA1217 | -2.37 |
| A_24_P684186 | NR_003955 | LOC647121 | embigin homolog (mouse) pseudogene | -2.37 |
| A_23_P133408 | NM_000758 | CSF2 | colony stimulating factor 2 (granulocyte-macrophage) | -2.36 |
| A_32_P138348 | NM_017527 | LY6K | lymphocyte antigen 6 complex, locus K" | -2.36 |
| A_23_P155755 | NM_002993 | CXCL6 | chemokine (C-X-C motif) ligand 6 (granulocyte chemotactic protein 2) | -2.33 |
| A_23_P71379 | NM_005672 | PSCA | prostate stem cell antigen | -2.32 |
| A_23_P101193 | NM_001080467 | MYO5B | myosin VB | -2.32 |
| A_23_P143713 | NM_021822 | APOBEC3G | apolipoprotein B mRNA editing enzyme, catalytic polypeptide-like 3G" | -2.32 |
| A_23_P52323 | NM_000494 | COL17A1 | collagen, type XVII, alpha 1" | -2.32 |
| A_24_P40721 | NM_018327 | SPTLC3 | serine palmitoyltransferase, long chain base subunit 3 | -2.32 |
| A_24_P142503 | NM_018242 | SLC47A1 | solute carrier family 47, member 1" | -2.31 |
| A_24_P81900 | NM_006931 | SLC2A3 | solute carrier family 2 (facilitated glucose transporter), member 3" | -2.31 |
| A_23_P376704 | NM_198289 | CIDEA | cell death-inducing DFFA-like effector a | -2.30 |
| A_24_P272146 | BC067092 | IGKC | immunoglobulin kappa constant | -2.30 |
| A_23_P161563 | NM_022337 | RAB38 | RAB38, member RAS oncogene family" | -2.29 |
| A_23_P121657 | NM_005114 | HS3ST1 | heparan sulfate (glucosamine) 3-O-sulfotransferase 1 | -2.27 |
| A_23_P48217 | NM_030817 | APOLD1 | apolipoprotein L domain containing 1 | -2.27 |
| A_23_P86012 | NM_001017402 | LAMB3 | laminin, beta 3" | -2.25 |
| A_23_P160968 | NM_018891 | LAMC2 | laminin, gamma 2" | -2.25 |
| A_23_P134734 | NM_017786 | GOLSYN | Golgi-localized protein | -2.24 |
| A_23_P217901 | NM_001126312 | RP11-544M22.4 | KAT protein | -2.22 |
| A_23_P88819 | NM_017458 | MVP | major vault protein | -2.21 |
| A_23_P111126 | L06175 | HCP5 | HLA complex P5 | -2.20 |
| A_23_P160720 | NM_018664 | BATF3 | basic leucine zipper transcription factor, ATF-like 3 | -2.20 |
| A_24_P173754 | NM_030806 | C1orf21 | chromosome 1 open reading frame 21 | -2.20 |
| A_24_P388786 | NM_001369 | DNAH5 | dynein, axonemal, heavy chain 5 | -2.19 |
| A_23_P407096 | NM_152625 | ZNF366 | zinc finger protein 366 | -2.19 |
| A_23_P131935 | NM_017671 | FERMT1 | fermitin family homolog 1 (Drosophila) | -2.18 |
| A_23_P381645 | NM_001005463 | EBF3 | early B-cell factor 3 | -2.16 |
| A_23_P125705 | NM_021963 | NAP1L2 | nucleosome assembly protein 1-like 2 | -2.16 |
| A_23_P422350 | NM_000260 | MYO7A | myosin VIIA | -2.15 |
| A_23_P398476 | NM_022658 | HOXC8 | homeobox C8 | -2.15 |
| A_23_P210581 | NM_002237 | KCNG1 | potassium voltage-gated channel, subfamily G, member 1" | -2.14 |
| A_32_P206415 | NM_001008781 | FAT3 | FAT tumor suppressor homolog 3 (Drosophila) | -2.13 |
| A_23_P149121 | NM_004675 | DIRAS3 | DIRAS family, GTP-binding RAS-like 3 | -2.13 |
| A_24_P944588 | NM_033196 | ZNF682 | zinc finger protein 682 | -2.12 |
| A_23_P171132 | NM_021783 | EDA2R | ectodysplasin A2 receptor | -2.12 |
| A_23_P104555 | NM_020349 | ANKRD2 | ankyrin repeat domain 2 (stretch responsive muscle) | -2.11 |
| A_23_P69206 | NM_003043 | SLC6A6 | solute carrier family 6 (neurotransmitter transporter, taurine), member 6" | -2.11 |
| A_23_P145054 | NM_001085480 | FAM162B | family with sequence similarity 162, member B" | -2.11 |
| A_23_P40217 | NM_018431 | DOK5 | docking protein 5 | -2.11 |
| A_23_P139123 | NM_000062 | SERPING1 | serpin peptidase inhibitor, clade G (C1 inhibitor), member 1" | -2.10 |
| A_24_P95059 | NM_007155 | ZP3 | zona pellucida glycoprotein 3 (sperm receptor) | -2.10 |
| A_23_P48438 | NM_199162 | ADPRHL1 | ADP-ribosylhydrolase like 1 | -2.10 |
| A_23_P94319 | NM_014867 | KBTBD11 | kelch repeat and BTB (POZ) domain containing 11 | -2.09 |
| A_23_P160214 | NM_001080494 | TTC39A | tetratricopeptide repeat domain 39A | -2.08 |
| A_24_P291231 | NM_016831 | PER3 | period homolog 3 (Drosophila) | -2.08 |
| A_23_P101623 | NM_022103 | ZNF667 | zinc finger protein 667 | -2.08 |
| A_23_P207221 | NM_018242 | SLC47A1 | solute carrier family 47, member 1" | -2.06 |
| A_24_P81789 | NM_019034 | RHOF | ras homolog gene family, member F (in filopodia) | -2.06 |
| A_23_P85952 | NM_024901 | DENND2D | DENN/MADD domain containing 2D | -2.03 |
| A_23_P16953 | NM_000867 | HTR2B | 5-hydroxytryptamine (serotonin) receptor 2B | -2.03 |
| A_23_P76538 | NM_017899 | TESC | tescalcin | -2.03 |
| A_23_P381431 | NM_030769 | NPL | N-acetylneuraminate pyruvate lyase (dihydrodipicolinate synthase) | -2.03 |
| A_23_P145024 | NM_000024 | ADRB2 | adrenergic, beta-2-, receptor, surface" | -2.03 |
| A_23_P169351 | NM_003026 | SH3GL2 | SH3-domain GRB2-like 2 | -2.02 |
| A_23_P152047 | NM_138967 | SCAMP5 | secretory carrier membrane protein 5 | -2.02 |
| A_23_P388168 | NM_002867 | RAB3B | RAB3B, member RAS oncogene family" | -2.02 |
| A_23_P201497 | NM_182663 | RASSF5 | Ras association (RalGDS/AF-6) domain family member 5 | -2.01 |
| A_24_P925342 | AB209275 | MAN1C1 | mannosidase, alpha, class 1C, member 1 | -2.00 |
| A_23_P130376 | NM_022068 | FAM38B | family with sequence similarity 38, member B | -2.00 |
