# Supplementary material for: A Stable and Reproducible Human Blood-Brain Barrier Model Derived from Hematopoietic Stem Cells
Source: PLoS One. 2014 Jun 17;9(6):e99733. doi: 10.1371/journal.pone.0099733 (PMC4061029; doi:10.1371/journal.pone.0099733)
Supplement: Table S4 — Up-regulated genes in the microarray. Gene expression on CD34+-derived ECs in co-culture at day 6 and 3 was significantly different regarding BBB markers, specifically for efflux transporters including solute carrier family members SLC30A3, SLC26A10, SLC13A3 and SLC44A5 (upregulated at day 6) and non-BBB markers such as channels and extracellular matrix. (DOC) [file pone.0099733.s007.doc]

**Table S4-** Up-regulated genes in the microarray. Gene expression on CD34^+^-derived ECs in co-culture at day 6 and 3 was significantly different regarding BBB markers, specifically for efflux transporters including solute carrier family members SLC30A3, SLC26A10, SLC13A3 and SLC44A5 (upregulated at day 6) and non-BBB markers such as channels and extracellular matrix.

|  |  |  | Co-culture 6 days versus Mono-culture 6 days |  |
| --- | --- | --- | --- | --- |
| Unique ID | Target ID | Gene Symbol | Gene Name | M Value |
| A_24_P940694 | AK091400 | SLC44A5 | solute carrier family 44, member 5 | 2.00 |
| A_24_P171141 | AF130049 | LOC114227 | hypothetical protein LOC114227 | 2.01 |
| A_23_P393401 | NR_003610 | PDXDC2 | pyridoxal-dependent decarboxylase domain containing 2 | 2.01 |
| A_23_P80570 | NM_001086 | AADAC | arylacetamide deacetylase | 2.02 |
| A_23_P81721 | NM_004277 | SLC25A27 | solute carrier family 25, member 27" | 2.03 |
| A_24_P419087 | NM_006576 | AVIL | advillin | 2.03 |
| A_23_P360542 | NR_023925 | C18orf2 | chromosome 18 open reading frame 2 | 2.04 |
| A_23_P346048 | NR_002824 | HERC2P2 | hect domain and RLD 2 pseudogene 2 | 2.04 |
| A_23_P60811 | NM_006252 | PRKAA2 | protein kinase, AMP-activated, alpha 2 catalytic subunit" | 2.05 |
| A_24_P222237 | NR_002824 | HERC2P2 | hect domain and RLD 2 pseudogene 2 | 2.07 |
| A_23_P207493 | NM_016424 | CROP | cisplatin resistance-associated overexpressed protein | 2.07 |
| A_32_P110550 | AB042555 | PDE4DIP | phosphodiesterase 4D interacting protein | 2.07 |
| A_23_P340308 | NM_176888 | TAS2R48 | taste receptor, type 2, member 48 | 2.08 |
| A_24_P60217 | AK055730 | SLC23A3 | solute carrier family 23 (nucleobase transporters), member 3" | 2.08 |
| A_24_P940725 | AL080186 | SFRS18 | splicing factor, arginine/serine-rich 18 | 2.08 |
| A_24_P98161 | NM_194455 | KRIT1 | KRIT1, ankyrin repeat containing | 2.09 |
| A_23_P28246 | NM_144712 | SLC23A3 | solute carrier family 23 (nucleobase transporters), member 3" | 2.09 |
| A_23_P54447 | BC069765 | C15orf5 | chromosome 15 open reading frame 5 | 2.10 |
| A_24_P453855 | AK126267 | PNPLA7 | patatin-like phospholipase domain containing 7 | 2.11 |
| A_32_P169491 | AK098200 | LOC161527 | hypothetical protein LOC161527 | 2.11 |
| A_24_P341985 | NM_031938 | BCO2 | beta-carotene oxygenase 2 | 2.11 |
| A_32_P468289 | AL162056 | DOPEY1 | dopey family member 1 | 2.12 |
| A_24_P50368 | NM_001001786 | BLID | BH3-like motif containing, cell death inducer" | 2.12 |
| A_24_P928522 | AK025142 | DST | dystonin | 2.13 |
| A_32_P216872 | BX647358 | PDXDC2 | pyridoxal-dependent decarboxylase domain containing 2 | 2.13 |
| A_23_P303851 | NM_176886 | TAS2R45 | taste receptor, type 2, member 45 | 2.13 |
| A_32_P211080 | NR_002824 | HERC2P2 | hect domain and RLD 2 pseudogene 2 | 2.14 |
| A_23_P75071 | NM_016195 | KIF20B | kinesin family member 20B | 2.15 |
| A_23_P354308 | AK025204 | ABI3BP | ABI family, member 3 (NESH) binding protein" | 2.15 |
| A_24_P8200 | AB095943 | SHPRH | SNF2 histone linker PHD RING helicase | 2.16 |
| A_24_P93754 | AB018323 | JMJD2C | jumonji domain containing 2C | 2.17 |
| A_23_P257164 | NM_000481 | AMT | aminomethyltransferase | 2.17 |
| A_32_P139738 | NR_002827 | HERC2P4 | hect domain and RLD 2 pseudogene 4 | 2.17 |
| A_24_P925158 | D26122 | SF1 | splicing factor 1 | 2.17 |
| A_32_P78385 | BC094802 | DPY19L2P2 | dpy-19-like 2 pseudogene 2 (C. elegans) | 2.17 |
| A_23_P306511 | BC022302 | CMAH | cytidine monophosphate-N-acetylneuraminic acid hydroxylase (CMP-N-acetylneuraminate monooxygenase) pseudogene | 2.19 |
| A_23_P36865 | NM_025114 | CEP290 | centrosomal protein 290kDa | 2.20 |
| A_32_P48244 | ENST00000358296 | ZNF100 | zinc finger protein 100 | 2.22 |
| A_24_P932416 | NM_001123228 | TMEM14E | transmembrane protein 14E | 2.23 |
| A_23_P31681 | AK074467 | C8orf38 | chromosome 8 open reading frame 38 | 2.23 |
| A_23_P8961 | NM_000880 | IL7 | interleukin 7 | 2.23 |
| A_32_P195850 | NM_173812 | DPY19L2 | dpy-19-like 2 (C. elegans) | 2.24 |
| A_24_P910580 | NM_181077 | GOLGA8A | golgi autoantigen, golgin subfamily a, 8A" | 2.26 |
| A_24_P463973 | AK123878 | MEG3 | maternally expressed 3 (non-protein coding) | 2.27 |
| A_32_P75559 | AK303593 | BST2 | bone marrow stromal cell antigen 2 | 2.27 |
| A_23_P253622 | AK024934 | ANKRD36B | ankyrin repeat domain 36B | 2.29 |
| A_23_P340312 | NM_176888 | TAS2R48 | taste receptor, type 2, member 48 | 2.30 |
| A_23_P115192 | NM_031282 | FCRL4 | Fc receptor-like 4 | 2.31 |
| A_24_P51067 | NM_025114 | CEP290 | centrosomal protein 290kDa | 2.31 |
| A_24_P265177 | AK022791 | PHC3 | polyhomeotic homolog 3 (Drosophila) | 2.32 |
| A_32_P156373 | XM_001715393 | LOC100132218 | hypothetical protein LOC100132218 | 2.33 |
| A_32_P208733 | AK055279 | UTP23 | UTP23, small subunit (SSU) processome component, homolog (yeast)" | 2.33 |
| A_24_P114249 | NM_004482 | GALNT3 | UDP-N-acetyl-alpha-D-galactosamine:polypeptide N-acetylgalactosaminyltransferase 3 (GalNAc-T3) | 2.36 |
| A_23_P374250 | NM_173812 | DPY19L2 | dpy-19-like 2 (C. elegans) | 2.37 |
| A_24_P687305 | NR_024583 | DKFZp434K191 | POM121 membrane glycoprotein-like 1 pseudogene | 2.38 |
| A_23_P426305 | NM_003734 | AOC3 | amine oxidase, copper containing 3 (vascular adhesion protein 1)" | 2.40 |
| A_23_P331072 | BX647210 | LRRIQ3 | leucine-rich repeats and IQ motif containing 3 | 2.43 |
| A_23_P125748 | NM_032441 | ZMAT1 | zinc finger, matrin type 1" | 2.44 |
| A_23_P37623 | NM_181077 | GOLGA8A | golgi autoantigen, golgin subfamily a, 8A" | 2.45 |
| A_24_P128442 | NM_152380 | TBX15 | T-box 15 | 2.48 |
| A_32_P776863 | NM_173649 | C2orf61 | chromosome 2 open reading frame 61 | 2.50 |
| A_23_P52121 | NM_002614 | PDZK1 | PDZ domain containing 1 | 2.50 |
| A_23_P253524 | NM_001813 | CENPE | centromere protein E, 312kDa" | 2.54 |
| A_23_P385911 | ENST00000396791 | KIAA1712 | KIAA1712 | 2.60 |
| A_23_P124805 | AK001243 | VPS13C | vacuolar protein sorting 13 homolog C (S. cerevisiae) | 2.62 |
| A_32_P122136 | AK057596 | LOC150759 | hypothetical protein LOC150759 | 2.67 |
| A_24_P246841 | NM_004277 | SLC25A27 | solute carrier family 25, member 27" | 2.77 |
| A_23_P23611 | NM_001008219 | AMY1C | amylase, alpha 1C (salivary) | 2.78 |
| A_23_P353614 | NM_152765 | C8orf46 | chromosome 8 open reading frame 46 | 2.80 |
| A_24_P916797 | AK000270 | AKAP9 | A kinase (PRKA) anchor protein (yotiao) 9 | 2.83 |
| A_24_P303420 | AK126092 | LOC221442 | hypothetical LOC221442 | 2.84 |
| A_24_P391591 | AK057596 | LOC150759 | hypothetical protein LOC150759 | 2.87 |
| A_24_P11100 | NM_032441 | ZMAT1 | zinc finger, matrin type 1" | 2.88 |
| A_23_P51587 | NM_002924 | RGS7 | regulator of G-protein signaling 7 | 2.95 |
| A_23_P414793 | NM_000096 | CP | ceruloplasmin (ferroxidase) | 3.00 |
| A_24_P85258 | NM_001080484 | KIAA1751 | KIAA1751 | 3.04 |
| A_24_P344890 | AK095605 | AMY2B | amylase, alpha 2B (pancreatic) | 3.05 |
| A_23_P302060 | NM_176891 | IFNE | interferon, epsilon" | 3.09 |
| A_24_P53595 | NM_016592 | GNAS | GNAS complex locus | 3.20 |
| A_24_P310256 | NM_139284 | LGI4 | leucine-rich repeat LGI family, member 4" | 3.32 |
| A_24_P332081 | AL832756 | JAKMIP3 | janus kinase and microtubule interacting protein 3 | 3.34 |
| A_23_P203191 | NM_000039 | APOA1 | apolipoprotein A-I | 3.40 |
| A_24_P341000 | AK092698 | FLJ35379 | similar to Alu subfamily J sequence contamination warning entry | 3.60 |
| A_32_P9941 | NM_007191 | WIF1 | WNT inhibitory factor 1 | 3.88 |
| A_32_P197561 | NM_024007 | EBF1 | early B-cell factor 1 | 4.42 |

|  |  |  | Co-culture 6 days versus Co-culture 3 days |  |
| --- | --- | --- | --- | --- |
| Unique ID | Target ID | Gene Symbol | Gene Name | M Value |
| A_24_P500584 | NR_001564 | XIST | X (inactive)-specific transcript (non-protein coding) | 12.60 |
| A_23_P155786 | NM_005420 | SULT1E1 | sulfotransferase family 1E, estrogen-preferring, member 1" | 8.20 |
| A_23_P95790 | NM_017625 | ITLN1 | intelectin 1 (galactofuranose binding) | 8.02 |
| A_23_P60130 | NM_052886 | MAL2 | mal, T-cell differentiation protein 2" | 7.51 |
| A_32_P179138 | NM_001130683 | GUCY1A3 | guanylate cyclase 1, soluble, alpha 3" | 6.65 |
| A_23_P350005 | NM_173553 | TRIML2 | tripartite motif family-like 2 | 5.61 |
| A_23_P43164 | NM_015170 | SULF1 | sulfatase 1 | 5.59 |
| A_24_P213161 | NM_017852 | NLRP2 | NLR family, pyrin domain containing 2" | 5.55 |
| A_23_P129085 | NM_145658 | SPESP1 | sperm equatorial segment protein 1 | 5.30 |
| A_23_P69573 | NM_000856 | GUCY1A3 | guanylate cyclase 1, soluble, alpha 3" | 5.24 |
| A_24_P53778 | NM_080878 | ITLN2 | intelectin 2 | 5.23 |
| A_24_P75917 | NM_182568 | CCDC144B | coiled-coil domain containing 144B | 5.17 |
| A_32_P154911 | NM_175887 | PRR15 | proline rich 15 | 5.15 |
| A_23_P67847 | NM_024572 | GALNT14 | UDP-N-acetyl-alpha-D-galactosamine:polypeptide N-acetylgalactosaminyltransferase 14 (GalNAc-T14) | 4.88 |
| A_24_P13041 | NM_145307 | RTKN2 | rhotekin 2 | 4.68 |
| A_23_P155688 | NM_021114 | SPINK2 | serine peptidase inhibitor, Kazal type 2 (acrosin-trypsin inhibitor) | 4.67 |
| A_24_P288915 | AK093811 | CCDC144B | coiled-coil domain containing 144B | 4.64 |
| A_23_P371729 | NM_005266 | GJA5 | gap junction protein, alpha 5, 40kDa" | 4.63 |
| A_23_P15450 | NM_018286 | TMEM100 | transmembrane protein 100 | 4.50 |
| A_23_P36531 | NM_004616 | TSPAN8 | tetraspanin 8 | 4.40 |
| A_23_P403445 | NM_006569 | CGREF1 | cell growth regulator with EF-hand domain 1 | 4.24 |
| A_23_P56746 | NM_004460 | FAP | fibroblast activation protein, alpha" | 4.19 |
| A_24_P288890 | NM_181709 | FAM101A | family with sequence similarity 101, member A | 4.07 |
| A_23_P52410 | NM_145307 | RTKN2 | rhotekin 2 | 4.05 |
| A_23_P95029 | NM_021021 | SNTB1 | syntrophin, beta 1 (dystrophin-associated protein A1, 59kDa, basic component 1)" | 4.02 |
| A_23_P406385 | NM_153350 | FBXL16 | F-box and leucine-rich repeat protein 16 | 4.01 |
| A_23_P105144 | NM_020974 | SCUBE2 | signal peptide, CUB domain, EGF-like 2 | 4.01 |
| A_32_P200697 | NM_181709 | FAM101A | family with sequence similarity 101, member A | 3.97 |
| A_23_P215459 | NM_000501 | ELN | elastin | 3.93 |
| A_23_P58676 | NM_024563 | C5orf23 | chromosome 5 open reading frame 23 | 3.89 |
| A_23_P217917 | NM_147148 | GSTM4 | glutathione S-transferase mu 4 | 3.89 |
| A_32_P181077 | NM_203447 | DOCK8 | dedicator of cytokinesis 8 | 3.88 |
| A_23_P257649 | NM_002899 | RBP1 | retinol binding protein 1, cellular" | 3.86 |
| A_23_P253536 | NM_000908 | NPR3 | natriuretic peptide receptor C/guanylate cyclase C (atrionatriuretic peptide receptor C) | 3.80 |
| A_23_P69497 | NM_003278 | CLEC3B | C-type lectin domain family 3, member B" | 3.79 |
| A_23_P327451 | NM_000908 | NPR3 | natriuretic peptide receptor C/guanylate cyclase C (atrionatriuretic peptide receptor C) | 3.78 |
| A_23_P421401 | NM_002609 | PDGFRB | platelet-derived growth factor receptor, beta polypeptide" | 3.78 |
| A_23_P19369 | NM_017640 | LRRC16A | leucine rich repeat containing 16A | 3.73 |
| A_24_P164505 | BC098294 | FAM106A | family with sequence similarity 106, member A" | 3.71 |
| A_24_P639679 | AK095831 | SNORD123 | small nucleolar RNA, C/D box 123" | 3.66 |
| A_24_P369232 | NM_031455 | CCDC3 | coiled-coil domain containing 3 | 3.64 |
| A_23_P69738 | NM_023940 | RASL11B | RAS-like, family 11, member B | 3.57 |
| A_24_P738168 | ENST00000329798 | FREM3 | FRAS1 related extracellular matrix 3 | 3.55 |
| A_23_P31273 | NM_001635 | AMPH | amphiphysin | 3.51 |
| A_23_P132956 | NM_004181 | UCHL1 | ubiquitin carboxyl-terminal esterase L1 (ubiquitin thiolesterase) | 3.50 |
| A_23_P93737 | NM_004411 | DYNC1I1 | dynein, cytoplasmic 1, intermediate chain 1" | 3.47 |
| A_23_P121926 | NM_005410 | SEPP1 | selenoprotein P, plasma, 1" | 3.44 |
| A_23_P63736 | BC007394 | MGC16291 | hypothetical protein MGC16291 | 3.41 |
| A_23_P144911 | NM_152403 | EGFLAM | EGF-like, fibronectin type III and laminin G domains | 3.39 |
| A_23_P37727 | NM_002996 | CX3CL1 | chemokine (C-X3-C motif) ligand 1 | 3.39 |
| A_23_P49391 | NM_016212 | TP53TG3 | TP53 target 3 | 3.32 |
| A_23_P79251 | NM_014600 | EHD3 | EH-domain containing 3 | 3.30 |
| A_23_P386030 | AF028828 | SNTB1 | syntrophin, beta 1 (dystrophin-associated protein A1, 59kDa, basic component 1)" | 3.24 |
| A_23_P83098 | NM_000689 | ALDH1A1 | aldehyde dehydrogenase 1 family, member A1" | 3.21 |
| A_23_P61967 | NM_014469 | RBMXL2 | RNA binding motif protein, X-linked-like 2" | 3.20 |
| A_23_P47579 | NM_176822 | NLRP14 | NLR family, pyrin domain containing 14 | 3.19 |
| A_24_P212021 | NM_000851 | GSTM5 | glutathione S-transferase mu 5 | 3.18 |
| A_23_P83134 | NM_002048 | GAS1 | growth arrest-specific 1 | 3.17 |
| A_23_P79272 | NM_001003683 | PDE1A | phosphodiesterase 1A, calmodulin-dependent" | 3.16 |
| A_23_P66827 | AK021862 | FAM106A | family with sequence similarity 106, member A | 3.13 |
| A_24_P118196 | NM_001080393 | GLT8D4 | glycosyltransferase 8 domain containing 4 | 3.11 |
| A_23_P92983 | NM_017614 | BHMT2 | betaine-homocysteine methyltransferase 2 | 3.11 |
| A_32_P191840 | XM_933903 | LOC644662 | similar to hCG2042541 | 3.09 |
| A_32_P16007 | NM_207355 | POTEB | POTE ankyrin domain family, member B" | 3.06 |
| A_32_P60065 | NM_004101 | F2RL2 | coagulation factor II (thrombin) receptor-like 2 | 3.02 |
| A_23_P144718 | NM_004101 | F2RL2 | coagulation factor II (thrombin) receptor-like 2 | 3.00 |
| A_23_P407497 | NM_013959 | NRG1 | neuregulin 1 | 2.97 |
| A_24_P270728 | NM_001042483 | NUPR1 | nuclear protein 1 | 2.97 |
| A_23_P33326 | NM_000679 | ADRA1B | adrenergic, alpha-1B-, receptor" | 2.91 |
| A_23_P207003 | NM_004574 | 38231 | septin 4 | 2.91 |
| A_23_P420348 | NM_174981 | POTED | POTE ankyrin domain family, member D" | 2.90 |
| A_23_P167030 | NM_000316 | PTH1R | parathyroid hormone 1 receptor | 2.88 |
| A_23_P54144 | NM_001202 | BMP4 | bone morphogenetic protein 4 | 2.87 |
| A_23_P151805 | NM_006329 | FBLN5 | fibulin 5 | 2.87 |
| A_32_P471485 | BC025765 | RTKN2 | rhotekin 2 | 2.86 |
| A_23_P333029 | NM_173549 | C8orf47 | chromosome 8 open reading frame 47 | 2.86 |
| A_23_P26890 | NM_024302 | MMP28 | matrix metallopeptidase 28 | 2.85 |
| A_23_P56328 | NM_031310 | PLVAP | plasmalemma vesicle associated protein | 2.84 |
| A_32_P109214 | NM_001004306 | MGC87631 | similar to hypothetical protein FLJ36492 | 2.83 |
| A_23_P113351 | NM_004684 | SPARCL1 | SPARC-like 1 (hevin) | 2.83 |
| A_23_P155596 | NM_001002294 | FMO3 | flavin containing monooxygenase 3 | 2.83 |
| A_23_P114883 | NM_002023 | FMOD | fibromodulin | 2.82 |
| A_24_P220485 | NM_182487 | OLFML2A | olfactomedin-like 2A | 2.81 |
| A_24_P943588 | AF201385 | TXNRD2 | thioredoxin reductase 2 | 2.79 |
| A_24_P246196 | NM_214675 | CLEC4M | C-type lectin domain family 4, member M" | 2.78 |
| A_23_P302672 | NM_145244 | DDIT4L | DNA-damage-inducible transcript 4-like | 2.78 |
| A_23_P75769 | NM_024021 | MS4A4A | membrane-spanning 4-domains, subfamily A, member 4 | 2.78 |
| A_23_P64785 | NM_152320 | ZNF641 | zinc finger protein 641 | 2.77 |
| A_24_P292849 | AL137382 | LOC146429 | hypothetical protein LOC146429 | 2.77 |
| A_32_P50066 | NM_001039580 | MAP9 | microtubule-associated protein 9 | 2.76 |
| A_23_P144916 | NM_005110 | GFPT2 | glutamine-fructose-6-phosphate transaminase 2 | 2.75 |
| A_23_P422831 | NM_004816 | C9orf61 | chromosome 9 open reading frame 61 | 2.74 |
| A_23_P428080 | AB020701 | KIAA0894 | KIAA0894 protein | 2.74 |
| A_23_P302568 | NM_003459 | SLC30A3 | solute carrier family 30 (zinc transporter), member 3 | 2.73 |
| A_23_P214168 | NM_004370 | COL12A1 | collagen, type XII, alpha 1" | 2.72 |
| A_23_P122924 | NM_002192 | INHBA | inhibin, beta A" | 2.71 |
| A_23_P29124 | NM_002688 | 38596 | septin 5 | 2.71 |
| A_23_P360534 | NR_023925 | C18orf2 | chromosome 18 open reading frame 2 | 2.70 |
| A_23_P214803 | NM_014841 | SNAP91 | synaptosomal-associated protein, 91kDa homolog (mouse) | 2.70 |
| A_32_P32413 | NM_015559 | SETBP1 | SET binding protein 1 | 2.70 |
| A_24_P291814 | NM_004370 | COL12A1 | collagen, type XII, alpha 1" | 2.69 |
| A_24_P208436 | NM_001003683 | PDE1A | phosphodiesterase 1A, calmodulin-dependent | 2.67 |
| A_23_P51587 | NM_002924 | RGS7 | regulator of G-protein signaling 7 | 2.67 |
| A_23_P414793 | NM_000096 | CP | ceruloplasmin (ferroxidase) | 2.65 |
| A_23_P110473 | NM_004536 | NAIP | NLR family, apoptosis inhibitory protein" | 2.64 |
| A_23_P138655 | NM_057157 | CYP26A1 | cytochrome P450, family 26, subfamily A, polypeptide 1" | 2.64 |
| A_23_P215744 | NM_033427 | CTTNBP2 | cortactin binding protein 2 | 2.63 |
| A_24_P712271 | NM_207328 | LOC150763 | hypothetical protein LOC150763 | 2.63 |
| A_24_P221414 | NM_004411 | DYNC1I1 | dynein, cytoplasmic 1, intermediate chain 1" | 2.61 |
| A_23_P372974 | NM_152402 | TRAM1L1 | translocation associated membrane protein 1-like 1 | 2.61 |
| A_24_P222237 | NR_002824 | HERC2P2 | hect domain and RLD 2 pseudogene 2 | 2.60 |
| A_23_P116642 | NM_133489 | SLC26A10 | solute carrier family 26, member 10" | 2.60 |
| A_23_P87879 | NM_001781 | CD69 | CD69 molecule | 2.59 |
| A_23_P4551 | NM_015559 | SETBP1 | SET binding protein 1 | 2.59 |
| A_23_P8820 | NM_001442 | FABP4 | fatty acid binding protein 4, adipocyte" | 2.59 |
| A_32_P211080 | NR_002824 | HERC2P2 | hect domain and RLD 2 pseudogene 2 | 2.58 |
| A_23_P371495 | NM_175861 | TMTC1 | transmembrane and tetratricopeptide repeat containing 1 | 2.58 |
| A_24_P218805 | NM_017409 | HOXC10 | homeobox C10 | 2.57 |
| A_23_P121564 | NM_000857 | GUCY1B3 | guanylate cyclase 1, soluble, beta 3 | 2.56 |
| A_23_P126075 | NM_002245 | KCNK1 | potassium channel, subfamily K, member 1" | 2.55 |
| A_24_P356916 | NM_001011554 | SLC13A3 | solute carrier family 13 (sodium-dependent dicarboxylate transporter), member 3" | 2.54 |
| A_24_P53465 | NM_214675 | CLEC4M | C-type lectin domain family 4, member M" | 2.53 |
| A_23_P152305 | NM_001797 | CDH11 | cadherin 11, type 2, OB-cadherin (osteoblast)" | 2.52 |
| A_24_P184803 | NM_004086 | COCH | coagulation factor C homolog, cochlin (Limulus polyphemus)" | 2.50 |
| A_32_P34444 | NM_025135 | FHOD3 | formin homology 2 domain containing 3 | 2.50 |
| A_23_P357571 | NM_000854 | GSTT2 | glutathione S-transferase theta 2 | 2.49 |
| A_23_P346048 | NR_002824 | HERC2P2 | hect domain and RLD 2 pseudogene 2 | 2.48 |
| A_23_P346093 | NM_152468 | TMC8 | transmembrane channel-like 8 | 2.48 |
| A_23_P166797 | NM_022147 | RTP4 | receptor (chemosensory) transporter protein 4 | 2.47 |
| A_23_P419696 | NM_144586 | LYPD1 | LY6/PLAUR domain containing 1 | 2.47 |
| A_23_P204286 | NM_000900 | MGP | matrix Gla protein | 2.47 |
| A_32_P139738 | NR_002827 | HERC2P4 | hect domain and RLD 2 pseudogene 4 | 2.46 |
| A_23_P106933 | NM_052956 | ACSM1 | acyl-CoA synthetase medium-chain family member 1 | 2.45 |
| A_23_P904 | NM_024603 | BEND5 | BEN domain containing 5 | 2.44 |
| A_23_P115161 | NM_002036 | DARC | Duffy blood group, chemokine receptor" | 2.44 |
| A_23_P27013 | NM_024017 | HOXB9 | homeobox B9 | 2.43 |
| A_23_P142239 | AK027130 | LOC541469 | hypothetical protein LOC541469 | 2.41 |
| A_24_P221327 | BC020847 | LOC644246 | hypothetical protein LOC644246 | 2.40 |
| A_23_P28334 | NM_003853 | IL18RAP | interleukin 18 receptor accessory protein | 2.33 |
| A_23_P259442 | NM_001873 | CPE | carboxypeptidase E | 2.33 |
| A_23_P302634 | BC101016 | C12orf64 | chromosome 12 open reading frame 64 | 2.31 |
| A_23_P50697 | NM_006905 | PSG1 | pregnancy specific beta-1-glycoprotein 1 | 2.31 |
| A_24_P579826 | BC071681 | ARL17 | ADP-ribosylation factor-like 17 | 2.31 |
| A_23_P258769 | NM_002121 | HLA-DPB1 | major histocompatibility complex, class II, DP beta 1" | 2.31 |
| A_23_P52761 | NM_002423 | MMP7 | matrix metallopeptidase 7 (matrilysin, uterine)" | 2.31 |
| A_23_P257993 | NM_004944 | DNASE1L3 | deoxyribonuclease I-like 3 | 2.30 |
| A_23_P34126 | NM_001711 | BGN | biglycan | 2.30 |
| A_23_P19020 | NM_005460 | SNCAIP | synuclein, alpha interacting protein" | 2.29 |
| A_24_P331830 | NM_015209 | RP1-21O18.1 | kazrin | 2.29 |
| A_23_P337346 | AK056484 | hCG_2009921 | hypothetical locus LOC441204 | 2.28 |
| A_23_P13094 | NM_002425 | MMP10 | matrix metallopeptidase 10 (stromelysin 2) | 2.28 |
| A_23_P6818 | NM_020163 | SEMA3G | sema domain, immunoglobulin domain (Ig), short basic domain, secreted, (semaphorin) 3G | 2.28 |
| A_24_P128442 | NM_152380 | TBX15 | T-box 15 | 2.27 |
| A_24_P246406 | NM_001006605 | FAM69A | family with sequence similarity 69, member A" | 2.26 |
| A_23_P356494 | NM_006846 | SPINK5 | serine peptidase inhibitor, Kazal type 5" | 2.25 |
| A_24_P64401 | BC007394 | MGC16291 | hypothetical protein MGC16291 | 2.25 |
| A_24_P940694 | AK091400 | SLC44A5 | solute carrier family 44, member 5 | 2.25 |
| A_23_P206920 | NM_001040114 | MYH11 | myosin, heavy chain 11, smooth muscle | 2.24 |
| A_23_P16252 | NM_002257 | KLK1 | kallikrein 1 | 2.23 |
| A_24_P231829 | NM_017614 | BHMT2 | betaine-homocysteine methyltransferase 2 | 2.23 |
| A_24_P59799 | NM_024781 | CCDC102B | coiled-coil domain containing 102B | 2.22 |
| A_23_P109427 | NM_000854 | GSTT2 | glutathione S-transferase theta 2 | 2.21 |
| A_24_P245589 | NM_031310 | PLVAP | plasmalemma vesicle associated protein | 2.20 |
| A_23_P256033 | NM_001958 | EEF1A2 | eukaryotic translation elongation factor 1 alpha 2 | 2.19 |
| A_23_P357207 | NM_138409 | MRAP2 | melanocortin 2 receptor accessory protein 2 | 2.18 |
| A_32_P2452 | NM_175861 | TMTC1 | transmembrane and tetratricopeptide repeat containing 1 | 2.17 |
| A_23_P30075 | NM_006095 | ATP8A1 | ATPase, aminophospholipid transporter (APLT), class I, type 8A, member 1" | 2.17 |
| A_23_P423074 | NM_015566 | FAM169A | family with sequence similarity 169, member A" | 2.17 |
| A_24_P208595 | NM_053034 | ANTXR1 | anthrax toxin receptor 1 | 2.15 |
| A_24_P273799 | ENST00000301042 | ZNF641 | zinc finger protein 641 | 2.15 |
| A_23_P370027 | AK124788 | GGT7 | gamma-glutamyltransferase 7 | 2.14 |
| A_24_P231010 | NM_018995 | MOV10L1 | Mov10l1, Moloney leukemia virus 10-like 1, homolog (mouse)" | 2.13 |
| A_23_P204847 | NM_002298 | LCP1 | lymphocyte cytosolic protein 1 (L-plastin) | 2.13 |
| A_23_P116898 | NM_000014 | A2M | alpha-2-macroglobulin | 2.12 |
| A_32_P24122 | NM_015894 | STMN3 | stathmin-like 3 | 2.12 |
| A_24_P192485 | NM_002546 | TNFRSF11B | tumor necrosis factor receptor superfamily, member 11b" | 2.10 |
| A_23_P39202 | NM_033520 | C19orf33 | chromosome 19 open reading frame 33 | 2.10 |
| A_24_P396662 | NM_147148 | GSTM4 | glutathione S-transferase mu 4 | 2.09 |
| A_23_P122906 | NM_015570 | AUTS2 | autism susceptibility candidate 2 | 2.09 |
| A_23_P217319 | NM_004114 | FGF13 | fibroblast growth factor 13 | 2.08 |
| A_24_P380734 | NM_002998 | SDC2 | syndecan 2 | 2.08 |
| A_24_P363408 | NM_012259 | HEY2 | hairy/enhancer-of-split related with YRPW motif 2 | 2.07 |
| A_23_P44794 | NM_138453 | RAB3C | RAB3C, member RAS oncogene family" | 2.06 |
| A_23_P305198 | NM_003151 | STAT4 | signal transducer and activator of transcription 4 | 2.04 |
| A_23_P203957 | NM_175861 | TMTC1 | transmembrane and tetratricopeptide repeat containing 1 | 2.04 |
| A_23_P27795 | NM_021102 | SPINT2 | serine peptidase inhibitor, Kunitz type, 2" | 2.04 |
| A_23_P372308 | NM_020211 | RGMA | RGM domain family, member A" | 2.03 |
| A_32_P216566 | NM_001009994 | C6orf159 | chromosome 6 open reading frame 159 | 2.03 |
| A_24_P246573 | NM_015209 | RP1-21O18.1 | kazrin | 2.02 |
| A_23_P349321 | NM_022166 | XYLT1 | xylosyltransferase I | 2.01 |
| A_24_P920525 | AK022468 | SORBS1 | sorbin and SH3 domain containing 1 | 2.01 |
