# Supplementary material for: A Stable and Reproducible Human Blood-Brain Barrier Model Derived from Hematopoietic Stem Cells
Source: PLoS One. 2014 Jun 17;9(6):e99733. doi: 10.1371/journal.pone.0099733 (PMC4061029; doi:10.1371/journal.pone.0099733)
Supplement: Text S1 — Materials and Methods. (DOC) [file pone.0099733.s008.doc]

**Text S1. Materials and Methods**

**Isolation of pericytes.** Pericytes were extracted from freshly collected bovine brain capillaries . Brain capillaries were collected on a 60 µm nylon sieve (Blutex®, Saati, France) and suspended in Hanks Balanced Salt Solution (HBSS, Sigma-Aldrich) containing 10 mM HEPES and 0.1% BSA. This suspension was centrifuged at 1000g for 7 min at room temperature. The pellet was then digested with 2 mg/mL collagenase-dispase (Roche Diagnostics), 10 μg/mL DNaseI (Roche Diagnostics) and 0.147 μg/mL TLCK (Sigma-Aldrich), for 30 min at 37°C in a shaking water bath. After washes, the digested capillaries were seeded onto growth factor reduced Matrigel (BD Biosciences) -coated dishes (Corning) containing pericyte growth culture medium: DMEM (Life Technologies) supplemented with 20% fetal calf serum (Integro), 2 mM L-glutamine (Merck Chemicals), 50 µg/mL gentamicin (Biochrom AG) and 1 ng/mL bFGF (Sigma-Aldrich). The medium was changed every other day. Pericytes and ECs migrated from the vessels walls. Pericytes rapidly overgrew from capillaries and invaded the whole surface of the dishes. Confluent cultures consisting almost exclusively of pericytes were dissociated using trypsin/EDTA saline solution (0.05%/0.02% Biochrom AG) and cells were frozen in liquid nitrogen. For experiments, each pericyte vial was rapidly thawed and seeded in gelatin (Sigma-Aldrich) -coated 60-mm Petri dishes containing pericyte culture medium. After thawing, there were no ECs left in cultures. Pericytes were subcultured at a split ratio 1/3, and were used at passages ≤3.

**Rat glial cell cultures.** Primary cultures of glial cells were isolated from newborn rat cerebral cortex . After the meninges have been cleaned off, the brain tissue was forced gently through a nylon sieve. DMEM supplemented with 10% (v/v) FBS, 2 mM glutamine, and 50 μg/mL of gentamycin was used for the dissociation of cerebral tissue and development of glial cells. The glial cells were plated at a concentration of 5.5×10^4^ cells on 12-well plates. The medium was changed every second day. Three weeks after seeding, glial cultures were stabilized and composed of astrocytes (~60%), oligodendrocytes and microglial cells .

**Ultrastructural analysis of cell monolayers by transmission electron microscopy (TEM).** Wheat germ agglutinin conjugated horseradish peroxidase (WGAHRP) (Sigma-Aldrich) was used for ultrastructural analysis of EC monolayers. Filter inserts with ECs were transferred into plates containing 1.5 mL of HEPES-buffered Ringer’s solution (150 mM NaCl, 5.2 mM KCl, 2.2 mM CaCl_2_, 0.2 mM MgCl_2_-6H_2_O, 6 mM NaHCO_3_, 5 mM HEPES, 2.8 mM glucose, pH 7.4) (lower compartment), and 0.5 mL of HEPES-buffered Ringer’s solution supplemented with 0.1 mg/mL WGA-HRP was applied to the upper compartment. After 10 min incubation at 37°C in a 5% CO_2_/95% air atmosphere, the WGA-HRP solution was removed and the specimens were washed twice with HEPES-buffered Ringer’s solution and fixed for 1 h at room temperature with 2.5% glutaraldehyde and 1% paraformaldehyde in 0.1 M sodium cacodylate (pH 7.4). After washing with 0.1 M sodium cacodylate, the fixed EC monolayers were incubated for 30 min at room temperature with the HRP substrate 3, 3’-diaminobenzidine tetrahydrochloride (1.5 mg/mL; Sigma-Aldrich) and 0.02% H_2_O_2_ (v/v) in a TRIS-imidazol buffer (0.1 M imidazol, 0.05 M TRIS/HCl, pH 7.0). After washing with 0.1 M sodium cacodylate, cells were fixed again for 1 h at room temperature with 2.5% glutaraldehyde and 1% paraformaldehyde in cacodylate buffer. Specimens were washed twice with 0.1 M sodium cacodylate buffer, postfixed with 1% OsO4 in 0.1 M cacodylate buffer. After dehydration in graded ethanol, samples were embedded in Epon 812. Ultrathin sections were cut on Ultracut UCT (Leica), contrasted with uranyl acetate and lead citrate, and examined with a Jeol 1011 TEM at an accelerating voltage of 100 Kv.

**Reverse transcription and quantitative real time polymerase chain reaction (qRT-PCR) analysis**. CD34^+^-ECs cultured in different conditions were homogenized in Trizol reagent (Life Technologies) and total RNA was extracted using the RNeasy Mini Kit (Qiagen), according to manufacturer’s instructions. In all cases, cDNA was prepared from 1 μg total RNA using Taqman Reverse transcription reagents (Applied Biosystems). Non-quantitative RT-PCR was performed using the conditions described in and DNA migrated on a agarose gel electrophoresis (1.5%) with a low range DNA molecular weight marker (Euromedex) to visualize the sizes. Gels were then stained with gel red nucleic acid gel stain (Interchim) and visualized on a UV light transilluminator (Bio-Rad). Quantitative real time PCR (qRT-PCR) was performed using Power SYBR Green PCR Master Mix (Applied Biosystems) and the detection was carried out in a 7500 Fast Real-Time PCR System (Applied Biosystems). Quantification of target genes was performed relatively to the reference GAPDH gene: relative expression = 2^[-(Ct^_sample_^-Ct^_GADPH_)]. Primer sequences are given as supporting information (**Table S2**).

**Multidrug resistance accumulation assay.** Cell monolayers were washed with pre-warmed HEPES- buffered Ringer´s (RH) solution (NaCl 150 mM, KCl 5.2 mM, CaCl_2_ 2.2 mM, MgCl_2_ 0.2 mM, NaHCO_3_ 6 mM, Glucose 2.8 mM, HEPES 5 mM, water for injection). Cells were incubated with RH solution containing [^3^H]-vincristine sulphate at a final concentration of 66.5 nM with or without P-gp inhibitor (25 μM of verapamil (Sigma) or 0.5 μM elicridar). After 2 h, transwell filter with monolayer cells were placed on ice and the cells were washed five times with ice-cold RH solution. Cells were then lysed with 1% (v/v) Triton X-100 in RH solution for 5 min at 37°C and transferred to scintillation vials. Samples (100 μL) were diluted in liquid scintillation cocktail Ultima Gold M.V (4 mL, Perkin Elmer) and analyzed by a liquid scintillation analyzer, TRI-CARB 2100 TR (Perkin Elmer).

**Immunostaining.** Cells were fixed in cold methanol/acetone (50%/50% v/v) for 1 min or 4% (v/v) paraformaldehyde (Electron Microscopy Sciences, EMS) for 10 min at room temperature (see **Table S1**). After permeabilizing the cells with 0.1% (v/v) Triton X-100 (Sigma-Aldrich) for 5-10 min, whenever required, and blocking for 30 min with 1% (w/v) bovine serum albumin (BSA) solution (Sigma-Aldrich) or normal goat serum (10% (v/v), Sigma-Aldrich), the cells were incubated for 1 h with the primary monoclonal antibodies listed in **Table S1**, at room temperature. After washing, the cells were stained with a secondary antibody for 1 h in the dark at room temperature (**Table S1**). In each immunofluorescence experiment, an isotype-matched IgG control was used. The nuclei of cells was stained with 4',6-diamidino-2-phenylindole (DAPI; Sigma-Aldrich) or Hoescht reagent (ICN Pharmaceuticals). Cells were mounted using Mowiol (Sigma-Aldrich) containing an anti-fading agent (Dabco, Sigma-Aldrich) or cell-mounting medium from DAKO. Cells were examined with a Zeiss LSM 50 confocal microscope or with a Leica DMR fluorescence microscope (Leica Microsystems). In the last case, images were collected using a Cool SNAP RS Photometrics camera (Leica Microsystems) and were processed using Adobe Photoshop software 5.5 (Adobe systems).

**TEER measurements.** TEER (Ohm.cm²) of human ECs on Transwell filters was measured using the Millicell-ERS (Electrical Resistance System). The resistance of Matrigel-coated inserts was subtracted from the resistance obtained in the presence of the ECs according to the followed equation: TEER=[(TEER, cells)-(TEER, insert)×A], where A is the area of the filter (cm^2^).

***In vitro* free brain/plasma ratios**. The validation of brain homogenate binding and brain slice methods to determine fraction unbound brain (fu,br), which is subsequently multiplied by the total brain concentration to yield Cu,br have contributed to the acceptance of the ratio between the unbound concentrations in brain and plasma (Cu,br/Cu,pl) as a major pharmacokinetic parameter in drug discovery. The Cu,br/Cu,pl of Atenolol, Bupropion, Diazepam, Indomethacin, Lamotrigine, Levofloxacin, Methotrexate, Metoprolol and Verapamil has been determined using human BLECs and compared to human unbound CSF to plasma ratios (Cu,CSF/Cu,pl), taken as a surrogate measurement of Cu,br/Cu,pl in human. The human unbound CSF to plasma ratios were taken from the dataset published by Friden *et al.* . Atenolol, Bupropion, Diazepam, Metoprolol and Verapamil were kindly provided by AstraZeneca, Local Discovery Research Area CNS & Pain Control, (Södertälje, Sweden) at 10 mM in DMSO. Indomethacin, Lamotrigine, Levofloxacin, Methotrexate were kindly provided by SANOFI-AVENTIS Research & Development (Chilly-Mazarin, France) at 10 mM, in DMSO. Prior to experiments, rat glial cells were rinsed 3 times with RH solution (1.5 mL). Inserts with human brain-like endothelial cells were also rinsed and placed in rat glial cell wells. 0.5 mL of tested drugs at 2 µM in RH solution with 0.5% human serum albumin was added to the donor compartment. After 1 h of incubation, aliquots from the donor and receiver compartment were taken and analyzed (see below). The *in vitro* free brain/plasma ratios (Cu,b/Cu,p) were calculated using the free drug concentration in the receiver compartment and in the donor compartment after 1 h. These experimental data were computed into the blue-norna® brain exposure simulator ([http://www.blue-norna.com](http://www.blue-norna.com/)) to generate *in vitro* steady-state Cu,br/Cu,pl ratios.

All samples were analyzed using tandem mass spectrometry. Instruments that were used included: mass spectrometer, Quattro Premier XE (Waters); autosampler, Acquity sample manager; UPLC pump, Acquity Binary solvent manager (Waters); robot for sample preparation, Biomek FX (Beckman-Coulter). The following chemicals and reagents were used: ammonium acetate (Merck), acetonitrile gradient grade (Merck), methanol gradient grade (Merck), laboratory deionised water, further purified with a Milli-Q water purifying system and ammonium acetate 1 mol/L in Milli-Q water. Samples were stored in a freezer (-20°C). In order to minimize contamination of analysis instruments, protein precipitation was carried out on samples containing HSA; aliquots of samples were transferred to a deep well plate (1 mL), precipitated with acetonitrile and centrifuged (4000 rpm at 4 °C for 20 min). The supernatant was then transferred to a new deep well plate and RH buffer added. For chromatography the following system was used: analytical column, acquity UPLC BEH C18 1.7μm 2.1×30 mm (Waters); mobile phase A, 2% acetonitrile, 10 mM ammonium acetate and B, 80% acetonitrile in 10 mM ammonium acetate; gradient, 2% B for 0.2 min, 2-100% B in 0.3 min, held at 100% B for 0.2 min and returned to initial condition in one step; solvent delay 0.4 min, time between injections 1.5 min; flow rate 0.6 ml/min; loop: 10 µL; injection volume: 5-10 µL. The quantification of unknown samples was performed, using QuanLynx software. Response factors were constructed by plotting peak area of the analyte against concentration of each analyte using an average response factor of the donor (D0/C0) sample injections. The average RF function without weighting was used.

**Bidirectionnal transport assay.** Sodium fluorescein 1 µM or Cy3-human serum albumin 500 nM or Cy3-human immunoglobulin G 100 nM (Jackson ImmunoResearch) was applied on the apical or basolateral compartment of insert with ECs. The opposite compartment was filled with RH solution. After 120 min, the fluorescence was quantified on a Synergy H1 multiplate reader (Biotek) at an excitation/emission wavelength (nm) of 490/516 and 542/570 for sodium fluorescein and Cy3-human serum albumin/Cy3-human IgG, respectively. The efflux ratio was calculated using the equation: ER=(Papp,A>B)/Papp,B>A), where A>B and B>A denotes the transport direction in which P_app_ was determined. The apparent permeability coefficient (Papp) in cm/sec was calculated according to the following equation: Papp=(k×Vr)/(A×60), where k is the transport rate (min^-1^) defined as the slope obtained by linear regression of cumulative fraction absorbed (*FAcum*) as a function of time (min), V_r_ is the volume in the receiver chamber (cm^3^), and A is the area of the filter (cm^2^). Determination of the cumulative fraction absorbed (amount permeated), FAcum, versus time. FAcum was calculated from the equation: FAcum=ΣCRi/CDi, where CRi was the receiver concentration at the end of the interval i and CDi was the donor concentration at the beginning of interval i.

**FACS analysis.** Cells were dissociated from the culture plate by exposure to Cell Dissociation Buffer (Life Technologies) for 3-5 min and gentle pipetting, centrifuged and finally resuspended in PBS supplemented with 5% (v/v) FBS. The single cell suspensions were aliquoted (2.0×10^5^ cells per condition), fixed with 4% (v/v) paraformaldehyde (PFA; EMS) or ice-cold absolute methanol and permeabilized with 0.1% (w/v) Triton X-100 (Fluka) when necessary. The cells were stained with antigen-specific primary antibodies (dilution ratios and list of antibodies are given on **Table S1**). After the incubation with primary antibodies, cells were incubated with phycoerytrin (PE)-conjugated anti-rabbit (R&D Systems), and PE-conjugated anti-mouse (Santa Cruz) secondary antibodies. FACS Calibur (BD Biosciences) and BD Cell Quest Software (BD Biosciences) were used for the acquisition and analysis of the data.

**Western blot analysis.** Total protein was isolated from CD34^+^-ECs and pericytes in mono-culture or co-culture with RadioImmuno Precipitation Assay buffer [RIPA buffer; 50 mM Tris-HCl pH 7.4, 150 mM NaCl, 1% IGEPAL, 0.5% sodium deoxycholate, 0.1 % sodium dodecyl sulfate (SDS) and 1 mM ethylenediaminetetraacetic acid (EDTA)] supplemented with protease inhibitor cocktail (Sigma-Aldrich), 1 mM sodium orthovanadate (Sigma), 1 mM phenylmethanesulfonylfluoride (PMSF), 1 mM sodium fluoride (NaF) and 1 mM dithiothreitol (DTT). The protein samples were centrifuged at 14,000 g for 15 min at 4°C, the supernatants were collected into a new eppendorf tubes and stored at -20°C until use. Total protein (50 μg) was separated by 8-12.5% sodium dodecyl sulfate-polyacrylamide gel electrophoresis (SDS-PAGE) under reducing conditions and transferred to polyvinylidene difluoride (PVDF) membranes using the Trans-Blot® Turbo™ Transfer System (Bio-Rad). After blocking for 1 h at room temperature with PBS- 0.1% Tween® 20 (Sigma)-5% low fat milk, the membranes were incubated overnight at 4°C with antibodies against: Wnt3, Wnt7A, sonic hedgehog (Shh) (all from Santa Cruz Biotechnology), rabbit anti-β-Catenin total (Abcam) or α-tubulin (Sigma) followed by incubation with specific secondary antibodies for 1 h at room temperature (**Table S1**). The protein bands were revealed using enhanced chemiofluorescence [(ECF); GE Healthcare Life Sciences] reagent on the Biorad FX Molecular Imager (Bio-Rad).

**References**

1. Meresse S, Dehouck MP, Delorme P, Bensaid M, Tauber JP, et al. (1989) Bovine brain endothelial cells express tight junctions and monoamine oxidase activity in long-term culture. J Neurochem 53: 1363-1371.

2. Booher J, Sensenbrenner M (1972) Growth and cultivation of dissociated neurons and glial cells from embryonic chick, rat and human brain in flask cultures. Neurobiology 2: 97-105.

3. Descamps L, Coisne C, Dehouck B, Cecchelli R, Torpier G (2003) Protective effect of glial cells against lipopolysaccharide-mediated blood-brain barrier injury. Glia 42: 46-58.

4. Sano Y, Shimizu F, Abe M, Maeda T, Kashiwamura Y, et al. (2010) Establishment of a new conditionally immortalized human brain microvascular endothelial cell line retaining an in vivo blood-brain barrier function. J Cell Physiol 225: 519-528.

5. Kalvass JC, Maurer TS (2002) Influence of nonspecific brain and plasma binding on CNS exposure: implications for rational drug discovery. Biopharm Drug Dispos 23: 327-338.

6. Becker S, Liu X (2006) Evaluation of the utility of brain slice methods to study brain penetration. Drug Metab Dispos 34: 855-861.

7. Friden M, Gupta A, Antonsson M, Bredberg U, Hammarlund-Udenaes M (2007) In vitro methods for estimating unbound drug concentrations in the brain interstitial and intracellular fluids. Drug Metab Dispos 35: 1711-1719.

8. Friden M, Winiwarter S, Jerndal G, Bengtsson O, Wan H, et al. (2009) Structure-brain exposure relationships in rat and human using a novel data set of unbound drug concentrations in brain interstitial and cerebrospinal fluids. J Med Chem 52: 6233-6243.
